# Formation, stabilization and fate of acetaldehyde and higher aldehydes in an autonomously changing prebiotic system emerging from acetylene

**DOI:** 10.1038/s42004-023-00833-5

**Published:** 2023-02-22

**Authors:** Philippe Diederich, Thomas Geisberger, Yingfei Yan, Christian Seitz, Alexander Ruf, Claudia Huber, Norbert Hertkorn, Philippe Schmitt-Kopplin

**Affiliations:** 1 Helmholtz Munich, Research Unit Analytical BioGeoChemistry, Neuherberg, Germany; 2 Technical University of Munich Structural Membrane Biochemistry, BNMRZ, Lichtenbergstr 4, 85748 Garching, Germany; 3 Excellence Cluster ORIGINS, Boltzmannstraße 2, 85748 Garching, Germany; 4 Faculty of Physics, LMU Munich, Schellingstraße 4, 80799 Munich, Germany; 5 Technical University of Munich, Analytische Lebensmittel Chemie; Maximus-von-Forum 2, 85354 Freising, Germany; 6 Center for Astrochemical Studies, Max Planck Institute for Extraterrestrial Physics, Gießebachstraße 1, 85748 Garching bei München, Germany

**Keywords:** Biogeochemistry, Metals, Origin of life, Carbon cycle

## Abstract

Many essential building blocks of life, including amino acids, sugars, and nucleosides, require aldehydes for prebiotic synthesis. Pathways for their formation under early earth conditions are therefore of great importance. We investigated the formation of aldehydes by an experimental simulation of primordial early earth conditions, in line with the metal-sulfur world theory in an acetylene-containing atmosphere. We describe a pH-driven, intrinsically autoregulatory environment that concentrates acetaldehyde and other higher molecular weight aldehydes. We demonstrate that acetaldehyde is rapidly formed from acetylene over a nickel sulfide catalyst in an aqueous solution, followed by sequential reactions progressively increasing the molecular diversity and complexity of the reaction mixture. Interestingly, through inherent pH changes, the evolution of this complex matrix leads to auto-stabilization of de novo synthesized aldehydes and alters the subsequent synthesis of relevant biomolecules rather than yielding uncontrolled polymerization products. Our results emphasize the impact of progressively generated compounds on the overall reaction conditions and strengthen the role of acetylene in forming essential building blocks that are fundamental for the emergence of terrestrial life.

## Abstract (web summary)

Aldehydes can be formed under extraterrestrial conditions, however, the synthesis of aldehydes on early Earth remains largely unclear. Here, the authors report the formation of acetaldehydes and higher condensation products starting from a gaseous mixture of acetylene and carbon monoxide over nickel sulfide containing water with various pH-values under hydrothermal conditions.

## Introduction

Acetylene is a gaseous compound that was most likely available in the early Earth´s atmosphere and throughout the universe as it was formed through the photolysis of methane^1,2^. Spectroscopic measurements of astrophysical objects like Titan and Jupiter revealed acetylene as a part of their atmosphere^3^. Hypothetical scenarios for its role in prebiotic chemistry have already been discussed in the literature and mentioned routes to polycyclic aromatic hydrocarbons^4^ and other biomolecules^5^ under extraterrestrial conditions. At the same time, acetylene has been an often-used educt for numerous chemical reactions since the earliest stages of organic synthesis. After the discovery of acetylene hydration by Mikhail Kucherov in 1881^6^, acetylene became the main precursor for acetaldehyde production until modern industrial chemistry introduced the Wacker process^7^. Compounds carrying an aldehyde functional group are frequent educts in primordial synthesis pathways (and in chemical synthesis in general) as they are easily transformed into several coveted functional groups to fuel the origin of life^8^. The reactions of aldehydes are well characterized in the literature, making them an attractive basis for new reaction hypotheses^9^. Previous publications already reported the formation of acetaldehyde and various other aldehydes under extraterrestrial conditions, as extraterrestrial samples contain a large variety of organic compounds covering a broad chemical space^10^ whereas cometary ice contains acetaldehyde as a product of small building blocks and UV-radiation^11,12^. Ketones and aldehydes became part of systematic, targeted analytical screening in extraterrestrial materials (meteorites and return mission samples), underlining the importance of these compounds as essential reaction partners in synthesizing life-relevant molecules^13–16^. Despite the extraterrestrial hypothesis of aldehyde origins, the synthesis and accumulation of such precursors on early Earth remain largely unclear. In addition, the high reactivity of aldehydes leads to concentration issues and may prevent the accumulation of sufficient amounts of aldehydes that would allow specific reactions. Prebiotically relevant synthesis pathways, starting from aldehydes as biological building blocks, play a crucial role in understanding the origin of life. Synthesis of numerous necessary small building blocks of life has been demonstrated under varying levels of primordial plausibility^17^. As varying amounts of comparable chemical entities were also discovered in extraterrestrial materials such as meteorites or real samples from asteroid sample return missions^18–21^, the aim of this paper was a demonstration of a potentially universal, auto-regulatory, up-scalable synthesis of aldehydes explaining the occurrence of aldehydes under both Early Earth and extraterrestrial conditions.

The “metal-sulfur world” represents a prebiotically plausible hypothesis describing a reduced chemical environment allowing the emergence of life^22^. The investigation of this environment started with Wächtershäuser et al. with the incubation of CO and H_2_S in the presence of different metal-sulfur catalysts like FeS and NiS, which eventually resulted in activated acetic acid as an early analog of acetyl-CoA, hinting at a primitive form of the citric acid cycle^23^.

Recent work hypothesized acetylene as a precursor for reactions leading to relevant biomolecules, including unsaturated fatty acids and thiophens^24,25^. Until now, aldehydes were an unknown part of this hypothetical prebiotic environment, even though the potential relevance of acetaldehyde in the context of the metal sulfur world theory was already mentioned in a previous work^24^.

Here we provide experimental proof for the formation of acetaldehyde and a multitude of follow-up products. At the same time, we describe a pH-driven, intrinsic autoregulatory environment that concentrates acetaldehyde and changes the reaction path undertaken by aldehydes, fully compatible with early earth conditions and yielding sufficient amounts allowing for diversified detectable chemical reactions.

## Results

### Nickel sulfide catalyzes acetaldehyde formation

**Fig. 1:**
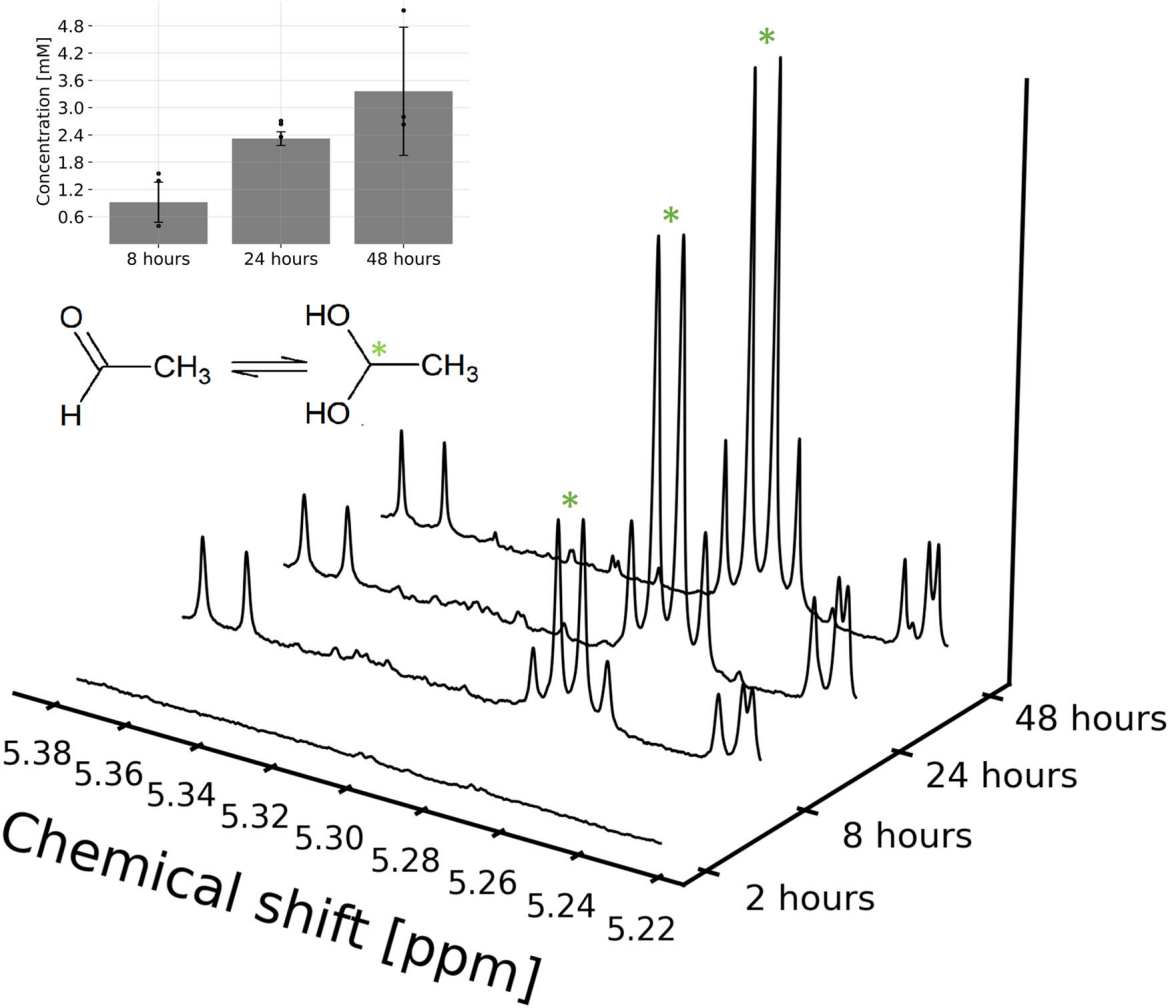
Quantification of acetaldehyde over time. 1D-^1^H spectrum showing the hydrogen signal of the single hydrogen connected to the carbon carrying the diol functionality (marked with a green asterisk). Spectra for four time points are shown. The bar plot shows the measured average concentration of acetaldehyde in the aqueous mixture over time, with error bars representing the standard deviation and black dots for individual data points.

To investigate the formation of acetaldehyde, we incubated a gaseous mixture of carbon monoxide and acetylene over water containing a nickel sulfide catalyst in an oxygen-free environment. This experimental setup was inspired by the acetylene metabolism of the anaerobic extant *Pelobacter acetylenicus* utilizing a tungstopterin enzyme to convert acetylene to acetaldehyde^26^. We determined the concentration of acetaldehyde via nuclear magnetic resonance spectroscopy (NMR) after different incubation periods (Fig. 1). The concentration of acetaldehyde showed a steady increase over two days, suggesting a higher conversion of acetylene to acetaldehyde compared to the reaction rate of acetaldehyde to later reaction products. Acetaldehyde reached a concentration of 3.3 mM after 48 h, representing a conversion of about 1% of the total available acetylene. Acetaldehyde could already be detected after 8 h of incubation, demonstrating the conversion efficiency with the used catalyst. Acetaldehyde results most likely from the hydration of acetylene followed by tautomerization of hydroxyethanal to acetaldehyde, analogous to the mercury salt catalyzed synthesis described by Kutscheroff in 1881. In this aqueous environment, acetaldehyde was detected as a mixture of the free aldehyde and its hydrate with a pH-dependent ratio. This hydration is, in our case, catalyzed by nickel sulfide, mimicking a mineral surface, as experiments without catalyst only resulted in trace amounts of acetaldehyde (<40 μM). Experiments with low concentrations of free nickel ions (4 µM), simulating the effect of dissolved NiS, didn’t lead to higher amounts of acetaldehyde compared to the experiments without NiS. However, experiments with trace amounts of sulfide (10 µM) lead to low but quantifiable acetaldehyde yields, suggesting a parallel pathway catalyzed by sulfide ions alone. This experiment led to concentrations of 360 µM of acetaldehyde, 10% of the yield with NiS (Supplementary Fig. 1). We performed additional experiments with FeS and NiS/FeS (1:1), as nickel sulfide is most often found in conjunction with iron sulfide in Nature. The setup with FeS alone did not show any detectable amount of free acetaldehyde nor the hydrate. The NiS/FeS setup produced acetaldehyde in the same concentration range as NiS alone (2.5 mM) (Supplementary Fig. 2), highlighting the importance of nickel sulfide in iron-nickel-sulfide minerals.

### Increased aldehyde diversity through aldol condensation

**Fig. 2:**
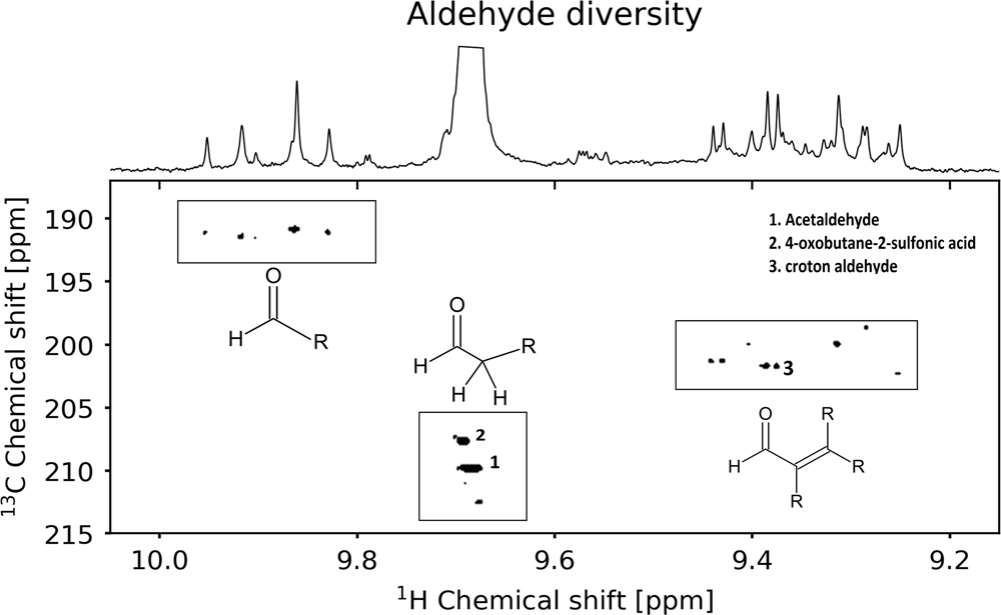
Aldehyde diversity formed after 7 days in an alkaline setup. The ^1^H,^13^C HSQC shows three different groups (marked with a black frame) of signals sharing similar hydrogen and carbon shifts, suggesting chemical similarity of the aldehyde group. The acetaldehyde signal is cut to allow magnification of the other aldehydes.

We detected trans-but-2-enal as the direct aldol condensation product of acetaldehyde, showing the feasibility of this type of reaction in the complex system, without interference from other matrix components. The aldol condensation and numerous other reactions drive the diversification of aldehydes in the explored system and generate a diverse aldehyde mixture (Fig. 2). The extensively investigated formose reaction generates a similar diversity of aldehydes through aldol condensation, starting with formaldehyde, leading to highly hydroxylated products, resulting in carbohydrates^27^. In contrast to the formose reaction, the condensation of acetaldehyde leads to highly reactive α,β-unsaturated aldehydes^28^. These aldehydes carry one double bond conjugated to the aldehyde group giving both the carbonyl carbon and the β-carbon an electrophilic character. This character allows nucleophiles to be added to the molecule. One of these potential nucleophiles is sulfur which will be discussed later. However, the aldol condensation reaction also carries the risk of rapid polymerization under favorable pH conditions. It leads to large, water-insoluble compounds, hereby removing the aldehydes from the reaction medium. The complex matrix of our system contains a large diversity of water-soluble aldehydes.

The numerous proton NMR resonances between 9.1 and 10 ppm reflect the diversity of aldehydes formed under these conditions. The observed clustering in the ^1^H,^13^C-HSQC experiment (an experiment that links hydrogen atom shifts to the carbon atom shift directly attached to the hydrogen atom) suggests a rough classification into three chemically similar aldehyde groups: α,β-unsaturated aldehydes (doublets 9.1–9.5 ppm), acetaldehyde (quartet 9.68 ppm), aliphatic aldehydes (multiplets 9.55–9.65 ppm) and aldehydes attached to an olefinic carbon without hydrogen or heteroatom, but not necessarily aromatic (singlets 9.75–10 ppm).

We further performed isotope-labeled experiments to clarify the origin of acetaldehyde in the investigated system and to confirm the hypothesized reactions. ^13^C-labeled acetylene experiments demonstrate acetylene as the sole origin of the detected aldehyde functional group. The absence of ^1^J_CH_ coupling in the ^1^H 1D NMR spectrum during ^13^C-labelling of carbon monoxide provides strong evidence for this hypothesis (Supplementary Fig. 3). Carbon monoxide does not react to detectable amounts of aldehydes under these conditions. Experiments with acetylene alone also produced acetaldehyde (Supplementary Fig. 4), excluding a relevant catalytical role of carbon monoxide in this reaction in form of nickel carbonyl for example.

### Increase in aldehyde diversity through Michael addition reactions with sulfur and formation of sulfuric acids

The overall complexity emerging out of the investigated system was already demonstrated by Fourier transform ion cyclotron resonance mass spectrometry measurements, showing an, until then, to the best of our knowledge unreported chemical space defined by sulfur^29^. These measurements revealed 2332 individual elemental compositions divided into 575 formulas consisting of only carbon, hydrogen, and oxygen and 1757 formulas with sulfur as an additional element. These results call for further characterization of the formed compounds and evaluation of their relevance to the origin of life.

The α,β-unsaturated functionality of the condensation products drives further diversification of aldehydes contained in the mixture by sequential aldol condensation and additions to the double bond by Michael addition reactions. Especially hydrogen sulfide and other thiols act as good nucleophiles and form new carbon-sulfur bonds. The sulfur in the investigated system originates from the NiS catalyst, as it is the only source of sulfur in the system. Sulfide addition is often observed in our system to be followed by the oxidation of sulfur to form a sulfonic acid group, shown through 4-oxobutane-2-sulfonic acid, which can be found in amounts comparable to acetaldehyde (half of the acetaldehyde concentration). More than 250 possibly sulfonated compounds could be detected by LC-MS, even though it remains unknown if all derive from Michael additions to α,β-unsaturated aldehydes (Supplementary Table 4). With the new knowledge of the existence of those sulfonic acids, a part of the sulfur compounds detected by FT-ICR-MS can be attributed to this compound class.

### Stabilization of aldehydes via intrinsic environmental pH changes

pH is a key parameter with a substantial impact on the reactivity of aldehydes. The pH of the investigated system evolves, starting at an alkaline pH of 12 and changing to a nearly neutral pH (7.6 ± 0.5 s.d.) *via* the formation of a large variety of carboxylic and sulfonic acids (Supplementary Table 1). The formation of highly unsaturated carboxylic acids has been described in a previous paper^24^. The chemistry leading to those carboxylic acids was already described by Walter Reppe in 1953^30^. The formation of these carboxylic acids requires carbon monoxide, also added to the gas phase of the experimental setup. However, smaller carboxylic acids like formic acid and acetic acid were also formed to some degree from acetylene alone. Formic acid was potentially formed through cleavage of the triple bond in acetylene by a so far unknown mechanism. This reaction usually requires strong oxidative conditions not present in the investigated setup^31^. This hypothesis was drawn from the observation of formic acid in setups with acetylene alone and the coexistence of labeled and unlabeled formic acids, in setups including labeled acetylene and unlabeled carbon monoxide (Supplementary Fig. 5). Acetic acid is observed and originates from acetylene alone^32^. We consider that the disproportionation of acetaldehyde provides the acetic acid to a certain extent, as ethanol is also detected in this prebiotic experiment. Acetaldehyde disproportionates into acetic acid and ethanol under all tested pH conditions. Lower pH (<8 after 7 days of incubation) enabled the stabilization of the observed aldehydes and slowed aldol condensation. The concentration of acetaldehyde was found to be 2.5 mM on average after 7 days. Experiments with added formic acid (pH 3) showed a significantly increased acetaldehyde yield (up to 40 mM, Supplementary Table 1). Further experiments with a standard acetaldehyde solution alone showed the compounds near inert behavior under acidic conditions. An incubation of acetaldehyde at 105 °C for one day in formic acid containing water (pH 4) did not lead to detectable reaction products besides a small amount of butenal (Supplementary Fig. 6). Morooka et al. reported the formation of lactic acid from formic acid and acetaldehyde under acidic conditions in subcritical water at 200–250 °C^33^. Even though both compounds were present in the mixture, lactic acid could not be detected under the much milder conditions utilized in this setup. Lower pH slows down the aldol condensation and inhibits rapid polymerization of the acetaldehyde, making it available for other reactions that are essential to the origin of life, depending on the prevailing pH.

### pH-dependent fate of the aldehydes: alkaline conditions

**Fig. 3:**
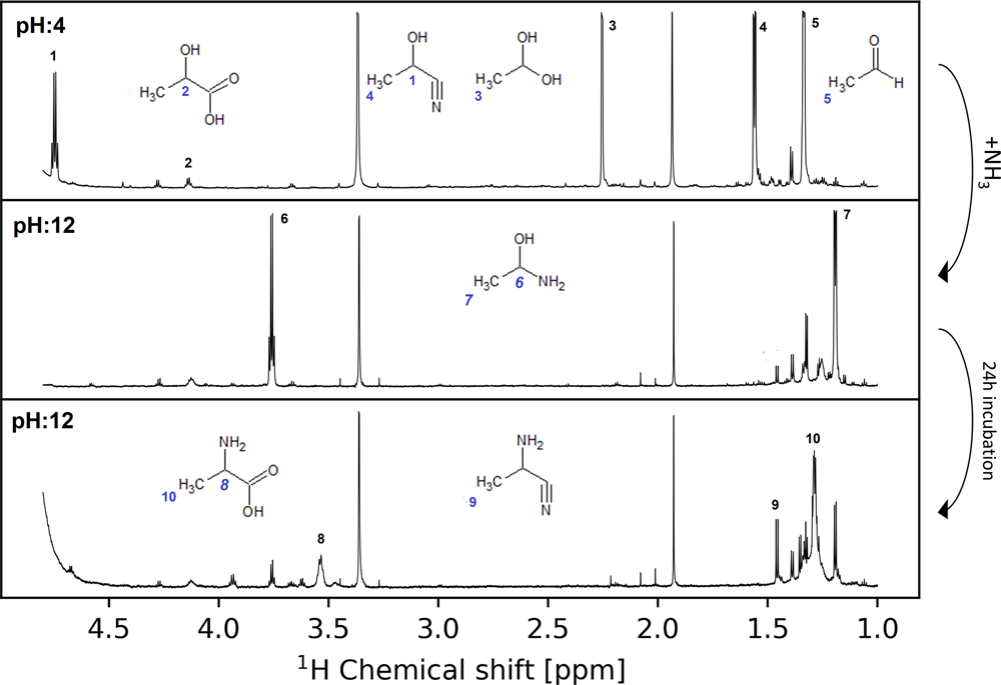
Investigation of the reactions of acetaldehyde under different pH conditions. The pH of an acidified setup was readjusted to alkaline conditions to observe the behavior of acetaldehyde. Blue numbers indicate detected hydrogen atoms and black numbers the corresponding resonance in the hydrogen spectrum.

To test the feasibility of a Strecker-type reaction in this complex mixture, we added potassium cyanide (KCN) and ammonium chloride (NH_4_Cl) to the alkaline setup. We detected the formation of alanine (180 µM, 0.02% yield based on ammonium chloride) in the resulting incubation mixture after 7 days. Alanine is the Strecker reaction product of acetaldehyde with potassium cyanide and ammonium chloride. The Strecker reaction is considered a likely reaction for forming amino acids under origin of life conditions^34^. Incubation of the same setup under acidic conditions (pH 3) leads to a mixture of acetaldehyde (27 mM) and 2-hydroxy propanenitrile (10.2 mM) but no alanine, showing the stabilization of the produced acetaldehyde and pH dependence of the fate of the formed aldehydes. The introduction of additional ammonia alkalinized the reaction mixture (pH 12), allowing the detection of intermediate products, namely 1-aminoethane-1-ol and 2-amino propanennitrile of the Strecker reaction from acetaldehyde to alanine in this chemically diverse environment (Fig. 3). These results support the hypothesis that the Strecker reaction is the main pathway leading to amino acid synthesis under these conditions from aldehydes. Alanine was additionally confirmed by LC-MS analysis. (Supplementary Table 3).

### pH-dependent fate of the aldehydes: acidic conditions

**Fig. 4:**
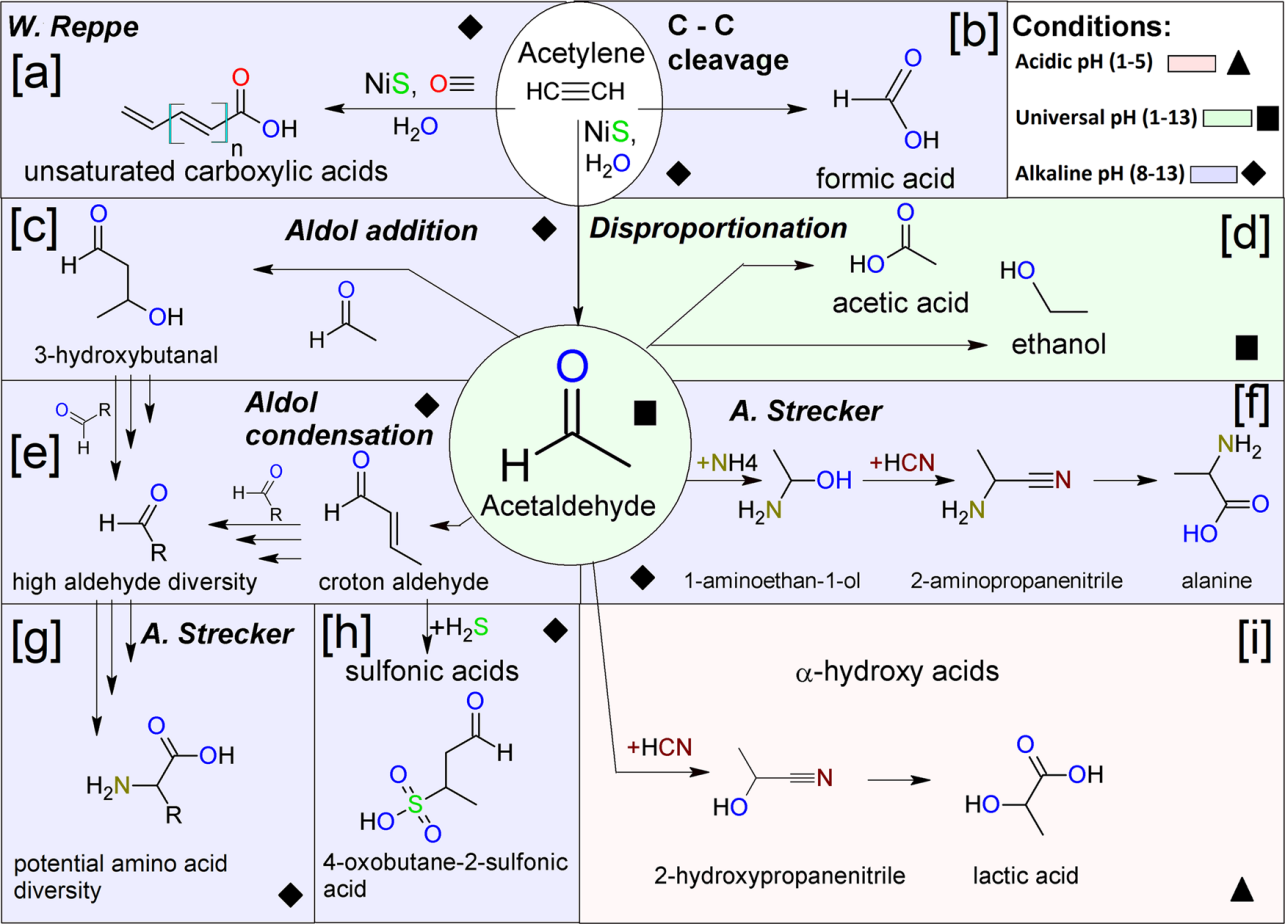
Reaction diagram showing all discussed reactions and detected compounds under different pH conditions. Background color and shapes indicate the pH conditions the compounds were detected at. The different panels show suggested formation pathways for the different compounds. Conversion of acetylene to unsaturated carboxylic acids via Reppe chemistry (**a**), conversion of acetylene into formic acid via an unkown pathway (**b**), condensation of acetaldehyde (c + e), dispropotionation of acetaldehyde leading to acetic acid and ethanol (**d**), Strecker reaction (f + g), michael addition followed by oxidation resulting in sulfonic acid (**h**), lactic acid formation (**i**).

Acidic conditions, occurring naturally through the generation of carboxylic and sulfonic acids, change the reaction pathway of aldehydes. A strong indicator of this behavior is the intermediate product 2-hydroxypropanenitrile, detected in the already mentioned setup acidified with formic acid. Formic acid was used for acidification as it is generated naturally in the investigated system. The acidified system should be thought of as a system that has already produced many carboxylic and sulfonic acids. The result is the conversion of acetaldehyde to an α-hydroxycarboxylic acid, namely lactic acid, instead of alanine via the hydrolysis of the cyanide group of the now prominent 2-hydroxypropanenitrile (Fig. 4, i). This pathway leading to lactic acid requires less harsh thermal conditions than the pathway starting from formic acid and acetaldehyde at 200–250 °C described by Morooka et al.^33^. It occurs naturally over time through the generation of carboxylic and sulfonic acids. To further validate the importance of acidic pH conditions, the pH of the acidified setup was further lowered with sulfonic acid to pH 1. After incubation, we observed a further increase in lactic acid concentration. α-hydroxy acids are known to promote peptide bond formation during dry-down reactions^35^.

An observed side effect of the lower pH is a higher percentage of dissolved NiS in the aqueous phase. This can be seen by the strong line broadening in the 1D-^1^H NMR spectra of the acidified solutions. The paramagnetic nickel in the solution leads to this effect. Carboxylic acids are strongly affected by a higher percentage of NiS, as they seem to coordinate with the Ni^2+^ in solution. At an acidic pH, the measurement became impossible without additional precipitation of the nickel ions with a sodium phosphate solution.

## Discussion

Our findings show an efficient and prebiotically relevant pathway to acetaldehyde and higher condensation products starting from a simple gaseous mixture of acetylene and carbon monoxide over NiS containing water under hydrothermal conditions. The mixture evolves by changing its pH in the first step by forming a multitude of carboxylic and sulfonic acids, which then, in a second step, changes the reaction pathway undertaken by the newly formed aldehydes. The aldehydes are later stabilized by the lowered pH of the complex mixture of carboxylic and sulfonic acids. Therefore, they stay available for further non-polymerization reactions even under high temperatures.

The first aldehyde formed in our experimental system, acetaldehyde, is described in the literature as required for multiple building blocks, including a path for synthesizing deoxyribonucleosides utilizing acetaldehyde as a critical educt^36^. Converting the aldehyde group into an amino acid group via the Strecker reaction is a more straightforward idea. The conditions of this primordial setup allow an expedient synthesis of amino acids, witnessed by the presence of alanine, readily formed *via* the Strecker reaction. The high diversity in aldehydes present in this system has therefore, the potential to form a diverse set of amino acids. The slow acidification of the environment by the carboxylic acids leads to favorable conditions for the Strecker reaction. We observe a transition from a basic pH, required for the nucleophilic addition of ammonia to the aldehyde group, to a more acidic pH that facilitates the imine formation and the hydrolysis of the α-aminonitrile.

Further acidification of the matrix leads to the detection of 2-hydroxy propanenitrile and lactic acid. These conditions suggest a pathway leading to α-hydroxycarboxylic acids via the hydrolysis of the nitrile group, analogous to the hydrolysis during the Strecker reaction^37^. The presence of the hydroxy group in alpha position enables this reaction only in an aged system that has already accumulated sufficient acids or lowered its pH in a different way. The positive impact of α-hydroxycarboxylic acids on peptide-bond formation during dry-down reactions is an interesting effect in a system that evolves from an amino acid-producing system to an α-hydroxycarboxylic acid producing system. The acidified environment also showed a higher concentration of dissolved nickel ions.

The α,β-unsaturated nature of a part of the formed aldehydes facilitates the functionalization of the formed compounds, as the addition of sulfur shows. The carbon-sulfur bond formation is a sought-after reaction in the field of the origin of life^38,39^. Early papers suggest a thioacid/thioester-based metabolism as a precursor to extant phosphate-based metabolic pathways^40,41^. The presence of sulfonic acids was also detected in the Murchison meteorite, even though a direct relevance for the origin-of-life remained uncommented^42^. Recent reports show the increased importance of sulfur functional groups, especially thiols, as precursors of protopeptides^43^ and organocatalysts^44^.

Future investigations could focus on a pH swing back to alkaline conditions due to external events. This alkalization would create starting conditions with much higher acetaldehyde concentrations, leading to stronger polymerization and higher-weight aldehydes. If the polymerization is stopped quickly enough by the formation of acids, this would change product distribution to potentially higher mass amino acids and sulfonic acids.

## Conclusion

Acetylene, a prominent molecule in the universe^45^, reported on the poles of Jupiter^46^, and supposed to be abundant on early Earth^47^, shows great potential as a precursor for primordial building blocks and reaction matrix modification. This paper sheds light on a possible pathway to acetaldehyde in a primordial setup aligned with the idea of a metal-sulfur world. The rapid diversification of acetaldehyde into a large variety of aldehydes and the transformation of acetaldehyde into alanine shows the potential to produce amino acids from acetylene gas in a one-pot experiment. Additionally, through the parallel formation of carboxylic acids and the efficient generation of sulfonic acids *via* Michael addition reactions, the environment intrinsically changes over time, especially the pH. This alteration changes the prevailing conditions to such an extent that the reactions involving the formed aldehydes shifted from an amino acid-producing system to an α-hydroxycarboxylic acid-producing system. Considering this change in reaction conditions, provoked by intrinsic, de novo compound synthesis within the same setup allowed us to demonstrate how this system autonomously changes its chemical output without any external input.

## Materials and methods

### Chemicals

A D_2_O sodium phosphate (for analysis, Merck KGaA) buffer (1.5 M) was prepared with added sodium trimethylsilylpropanoate (2 mM) (98 atom %D, Aldrich) as the internal standard. The pH of this buffer was adjusted to 7 with sodium hydroxide. This solution was used to reference and quantify signals in 1D-^1^H NMR experiments. Ammonia solution in water (25% ammonia, Lichropur Merck KGaA), Sulfuric acid (95–98%, Sigma-Aldrich), Acetaldehyde standard (ReagentPlus, >99.0%, Sigma-Aldrich)), Lactic acid standard (>99.0%, Sigma-Aldrich).

### Nitrogen-free bottles: setup S1

A 125 ml glass serum bottle was charged with 1.0 mM NiSO_4_ • 6 H_2_O (99%, Aldrich) and sealed with a silicon stopper. Three times the bottle was evacuated and filled with argon, finally ending in a deaerated state. Subsequently, the bottle was filled with 8.5 ml argon-saturated water, with 1.0 mL argon-saturated 1 M Na_2_S (solid Na_2_S: 99.99%, Sigma-Aldrich) solution with 0.5 mL 1 M NaOH solution and finally with 60 ml CO (2.44 mmol) and 60 ml (2.53 mmol) acetylene (acetone free), using gastight syringes for the injections. Reactions were carried out at 105 °C. After a reaction time of up to 7 days, the reaction mixture was allowed to cool down.

Acetylene and CO were replaced by argon in a blank run with otherwise identical composition.

### Nitrogen-free bottles: setup S1*

A 125 ml glass serum bottle was charged with 1.0 mM NiSO_4_ • 6 H_2_O (99%, Aldrich), 50 µl of formic acid and sealed with a silicon stopper. The bottle was evacuated Three times and filled with argon, finally ending in a deaerated state. Subsequently, the bottle was filled with 8.5 ml argon-saturated water, with 1.0 mL argon-saturated 1 M Na_2_S (solid Na_2_S: 99.99%, Sigma-Aldrich) solution with 0.5 mL 1 M NaOH solution and finally with 60 ml CO (2.44 mmol) and 60 ml (2.53 mmol) acetylene (acetone free), using gastight syringes for the injections. Reactions were carried out at 105 °C. After a reaction time of up to 7 days, the reaction mixture was allowed to cool down.

### Nitrogen-free ^13^C bottles setup

To confirm the hypothesized reactions, ^13^CO or ^13^C_2_-acetylene were used in representative experiments. Whereas ^13^CO (Cambridge Isotopes Laboratories Inc. (Tewksbury, MA, USA) could be used directly, ^13^C-acetylene had to be set free from Ethinyl-^13^C_2_-trimethylsilan (90 atom % ^13^C, Sigma-Aldrich). To release the ^13^C_2_acetylene from its silylated form, a glass serum bottle was charged with the stoichiometric amount of tetrabutylammonium fluoride (TBAF) (≥97.0%, SigmaAldrich). The bottle was sealed with a silicon stopper. The bottle was evacuated three times and flushed with argon, finally ending in a deaerated state. The ^13^C_2_-acetylene was transferred into the bottle using a gastight syringe. TBAF and ^13^C_2_-acetylene amounts were calculated to produce a slight overpressure in the serum bottle.

### Nitrogen-containing bottles: S2 setup

A 125 ml glass serum bottle was charged with 1.0 mM NiSO_4_ • 6 H_2_O (99%, Aldrich), 1.0 mmol NH_4_Cl (>99.5% Sigma-Aldrich) and 1.0 mmol KCN (>98.0 % Sigma-Aldrich) resulting in starting molarities of 100 mM for each nitrogen containing compound. and sealed with a silicon stopper. Three times the bottle was evacuated and filled with argon, finally ending in a deaerated state. Subsequently, the bottle was filled with 8.5 ml argon-saturated water, with 1.0 mL argon-saturated 1 M Na_2_S (solid Na_2_S: 99.99%, Sigma-Aldrich) solution with 0.5 mL 1 M NaOH solution and finally with 60 ml CO (2.44 mmol) and 60 ml (2.53 mmol) acetylene (acetone free), using gastight syringes for the injections. Reactions were carried out at 105 °C. After a reaction time of up to 7 days, the reaction mixture was allowed to cool down.

### Nitrogen-containing bottles: S2*

A 125 ml glass serum bottle was charged with 1.0 mM NiSO_4_ • 6 H_2_O (99%, Aldrich), 50 µl of formic acid, 1.0 mmol NH_4_Cl ( > 99.5% Sigma-Aldrich) and 1.0 mmol KCN (>98.0% Sigma-Aldrich) resulting in starting molarities of 100 mM for each nitrogen containing compound and sealed with a silicon stopper. Three times the bottle was evacuated and filled with argon, finally ending in a deaerated state. Subsequently, the bottle was filled with 8.5 ml argon-saturated water, with 1.0 mL argon-saturated 1 M Na_2_S (solid Na_2_S: 99.99%, Sigma-Aldrich) solution with 0.5 mL 1 M NaOH solution and finally with 60 ml CO (2.44 mmol) and 60 ml (2.53 mmol) acetylene (acetone free), using gastight syringes for the injections. Reactions were carried out at 105 °C. After a reaction time of up to 7 days, the reaction mixture was allowed to cool down.

### NMR spectroscopy

All NMR experiments were carried out on an 800 MHz Bruker AVANCE III spectrometer equipped with a 5 mm QCI-probe head at 300 K.

#### Quantification of acetaldehyde

1D-^1^H spectra were acquired for triplicates of bottles incubated for 8 h, 24 h, and 48 h. 150 µl supernatant of each centrifuged sample was spiked with 50 µl D_2_O buffer. The pulse program consisted of a simple 90° pulse followed by acquisition. 64 scans were acquired for each sample with a relaxation delay of 16 s and an acquisition time of 4 s. On-resonance pre-saturation was used during the relaxation delay to suppress the water signal. The optimized 90° pulse had a duration of 12.75 µs. Quantification was done by comparing the sum of the integrals after baseline correction of the hydrate single hydrogen signal and the acetaldehyde methyl hydrogens signal added together to the TSP internal reference integral. The average for every time point was calculated. The acquired FID was apodized with an exponential function (LB = 0.3), and Fourier transformed.

#### Aldehyde complexity

A bottle incubated for 7 days was used to prepare the sample. 180 µl of the sample were spiked with 20 µl of D_2_O buffer.

1D-^1^H spectrum was acquired using a 1D version of the nuclear Overhauser effect (NOE) experiment with on-resonance pre-saturation of the water signal during the relaxation delay of 6 s (s) and mixing time of 20 ms. 256 scans were acquired. HSQC spectrum was recorded with a phase-sensitive version using Echo/AntiechoTPPI gradient selection (hsqcetgpprsisp2.2), decoupling during acquisition, and onresonance pre-saturation during the relaxation delay (1.5 s). 197 increments were acquired with 800 transients each. The spectral width was set to 15 ppm in the F2 dimension and 40 ppm in the F1 dimension, with an offset of 200 ppm in the F1 dimension. The FID of the F2 dimension was apodized with a sine function (SSB = 2), and the F1 Dimension with a squared sine function (SSB = 3).

#### Alanine and lactic acid formation experiments

To observe the formation of alanine from acetaldehyde in the investigated system, bottle setup S2* was used as it showed a high amount of acetaldehyde due to the acidic conditions. 2 µl of ammonia solution (25%) was added to 200 µl of S2* solution contained in a 3 mm NMR tube with a final concentration of 140 mM. This reaction mixture was measured *via* NMR. A first 1D^-1^H spectrum was acquired immediately after the addition and a second after a 24 h incubation at 100 °C. Spectra were acquired with a simple 90-degree pulse (13.75 us) and on-resonance pre-saturation during the relaxation delay. Eight scans were acquired for each sample with a 4-second acquisition time and a relaxation delay of 16 s to allow for quantitative information.

The same experiment was repeated with sulfuric acid instead of ammonia to further prove the origin of lactic acid in this specific experimental setup.

Acquisition parameters for NMR experiments with the purpose of compound identification or spectra not shown in the main text can be found in Supplementary Table 2. LC-MS parameters can be found in Supplementary Methods page S14.

### pH-Measurement

The pH of the solutions was determined with a Metrohm Ecotrode Gel pH-Electrode (Mettler Toledo, Gießen, Germany) and checked with VWR chemicals Dosatest pH test strips pH 0-14 (VWR International, Radnor, Pennsylvania, USA).

## Supplementary information

Supplementary Information

Description of Additional Supplementary Files

Supplementary Data 1

## Data Availability

The authors declare that [the/all other] data supporting the findings of this study are available within the paper [and its supplementary information files]. Further data that support the findings of this study are available from the corresponding author upon reasonable request. All NMR spectra can be found in Supplementary Data 1
