# Supplementary material for: Formation, stabilization and fate of acetaldehyde and higher aldehydes in an autonomously changing prebiotic system emerging from acetylene
Source: Commun Chem. 2023 Feb 22;6:38. doi: 10.1038/s42004-023-00833-5 (PMC9947100; doi:10.1038/s42004-023-00833-5)
Supplement: Supplementary file 1 — Supplementary Information [file 42004_2023_833_MOESM1_ESM.pdf]

Supplementary Information

Formation, stabilization, and fate of acetaldehyde and higher aldehydes in an autonomously changing prebiotic system emerging from acetylene

Philippe Diederich [a], Thomas Geisberger [b], Yingfei Yan [a], Christian Seitz [b], Alexander Ruf [c,d], Claudia Huber [b], Norbert Hertkorn[a], Philippe Schmitt-Kopplin [a, e, f*]

[a] Helmholtz Munich, Research Unit Analytical BioGeoChemistry, Neuherberg, Germany.
[b] Technical University of Munich Structural Membrane Biochemistry, BNMRZ, Munich, Germany
[c] Excellence Cluster ORIGINS, Boltzmannstraße 2, 85748 Garching, Germany
[d] LMU Munich, Faculty of Physics, Schellingstraße 4, 80799 Munich, Germany

[e] Technical University of Munich, Analytische Lebensmittel Chemie; Maximus-von-Forum 2, 85354 Freising, Germany.

[f]Max Planck Institute for Extraterrestrial Physics, Center for Astrochemical Studies, Gießebachstraße 1, 85748 Garching bei München, Germany.

[*]Corresponding author: Research Unit Analytical BioGeoChemistry, Helmholtz Zentrum München–German Research Center for Environmental Health, Neuherberg, Germany

Email: schmitt-kopplin@helmholtz-muenchen.de

Supplementary methods

Experiments with trace amounts of dissolved ions

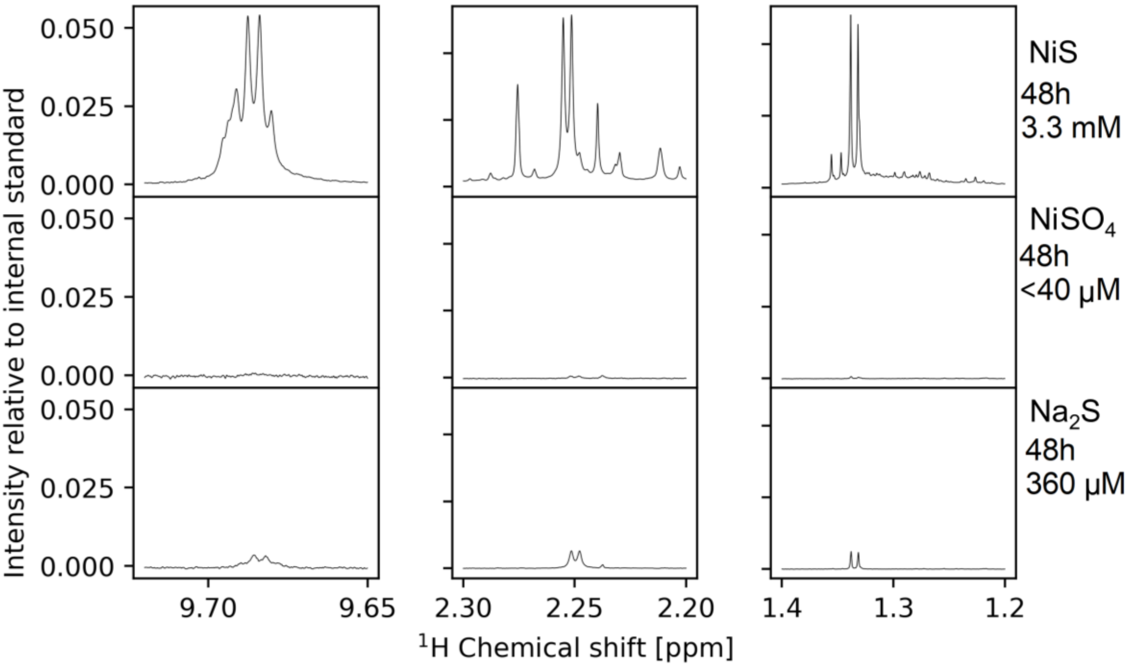

Supplementary Figure 1: 1D-^{1}H NMR spectra comparing the acetaldehyde yield of identical setups differing only in the potential catalyst. The NMR signals of the aldehyde hydrogen signal (column on the left), the methyl hydrogen of acetaldehyde (column in the middle), and the methyl hydrogen of the hydrate form of acetaldehyde (right column) are shown. The annotation on the right-hand side indicates the added catalyst, the incubation time, and the resulting yield in acetaldehyde.

Control bottles: setup S3_Supplement

A 125 ml glass serum bottle was charged with 1 mg $NiSO_4$ • 6 H_2O (99%, Aldrich) for the trace nickel experiment and with 10 µl of argon-saturated 1M Na_2S (solid Na_2S: 99.99%, Sigma-Aldrich) solution for the trace sulphide experiment. and sealed with a silicon stopper. Three times the bottle was evacuated and filled with argon, finally ending in a deaerated state. Subsequently, the bottle was filled with argon-saturated water (calculated for the end volume of 5 ml) with 1.0 mL 1M NaOH solution and finally with 60 ml CO (2.44 mmol) and 60 ml (2.53 mmol) acetylene (acetone free), using gastight syringes for the injections. Reactions were carried out at 105 °C. After a reaction time of up to 7 days, the reaction mixture was allowed to cool down. Acetylene and CO were replaced by argon in a blank run with otherwise identical composition.

Experiments with FiS and NiS/FeS

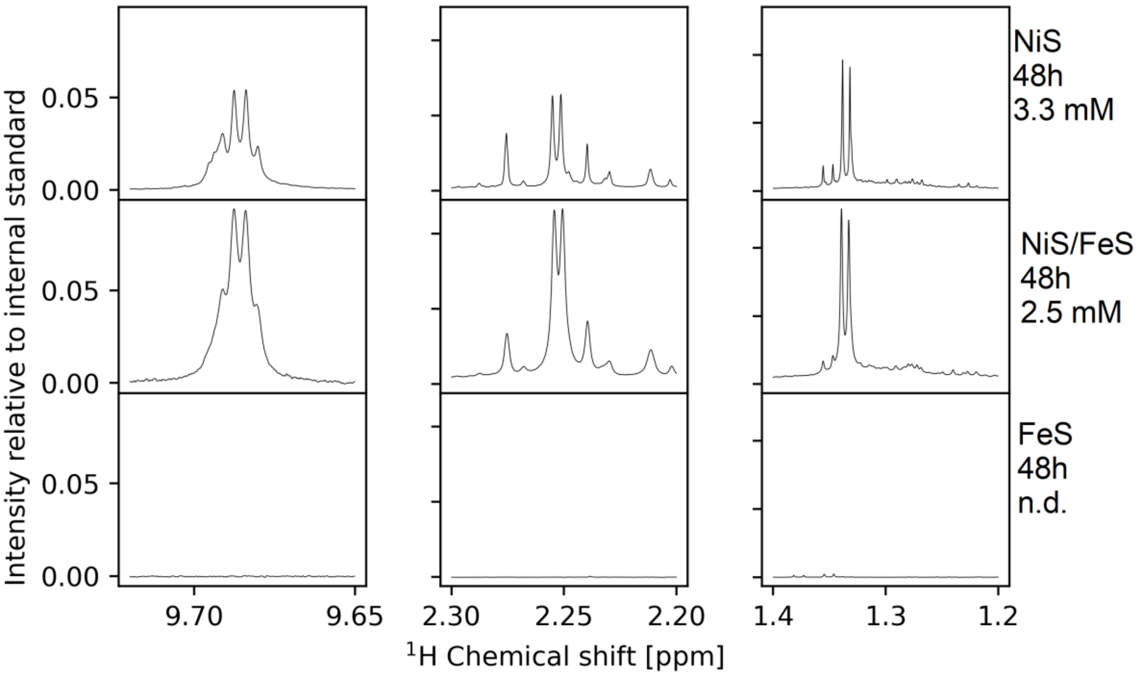

Supplementary Figure 2: Comparative acetaldehyde formation in setups with different catalyst compositions. The NMR signals of the aldehyde hydrogen signal (column on the left), the methyl hydrogen of acetaldehyde (column in the middle), and the methyl hydrogen of the hydrate form of acetaldehyde (right column) are shown. The annotation on the right-hand side indicates the added catalyst, the incubation time, and the resulting yield in acetaldehyde. The seemingly higher intensity in the NiS/FeS setup than the NiS setup stems purely from the broadened linewidth in the NiS/FeS setup and is an artifact of the processing. The concentrations derived from the peak integrals show the correct result.

Control bottles: setup S4_Supplement

A 125 ml glass serum bottle was charged with 131 mg $NiSO_4$ • 6 H_2O (99%, Aldrich) and 139 mg $FeSO_4$ • 7 H_2O (99%, Aldrich) for the NiS/FeS experiment and with 278 mg $FeSO_4$ • 7 H_2O (99%, Aldrich) for the FeS-only experiment. and sealed with a silicon stopper. Three times the bottle was evacuated and filled with argon, finally ending in a deaerated state. Subsequently, the bottle was filled with argon-saturated water (calculated for the end volume of 5 ml) with 1.0 mL argon-saturated 1M Na_2S (solid Na_2S: 99.99%, Sigma-Aldrich) solution with 1.0 mL 1M NaOH solution and finally with 60 ml CO (2.44 mmol) and 60 ml (2.53 mmol) acetylene (acetone free), using gastight syringes for the injections. Reactions were carried out at 105 °C. After a reaction time of up to 7 days, the reaction mixture was allowed to cool down. Acetylene and CO were replaced by argon in a blank run with otherwise identical composition.

Unlabelled versus labelled experiments

^{13}C labelling leads to additional splitting in 1D-^{1}H NMR spectra because of the NMR activity of this isotope. $^{1}J_{CH}$-couplings between the detected hydrogen signal and the ^{13}C-atom are 120-180 Hz. The absence of such a coupling in ^{13}CO-labelled experiments establishes acetylene as the origin of all detected aldehydes. On the other hand, experiments with ^{13}C-labelled acetylene showed the mentioned $^{1}J_{CH}$-splittings. $^{1}J_{CH}$ and $^{2}J_{CH}$ couplings were clearly observed.

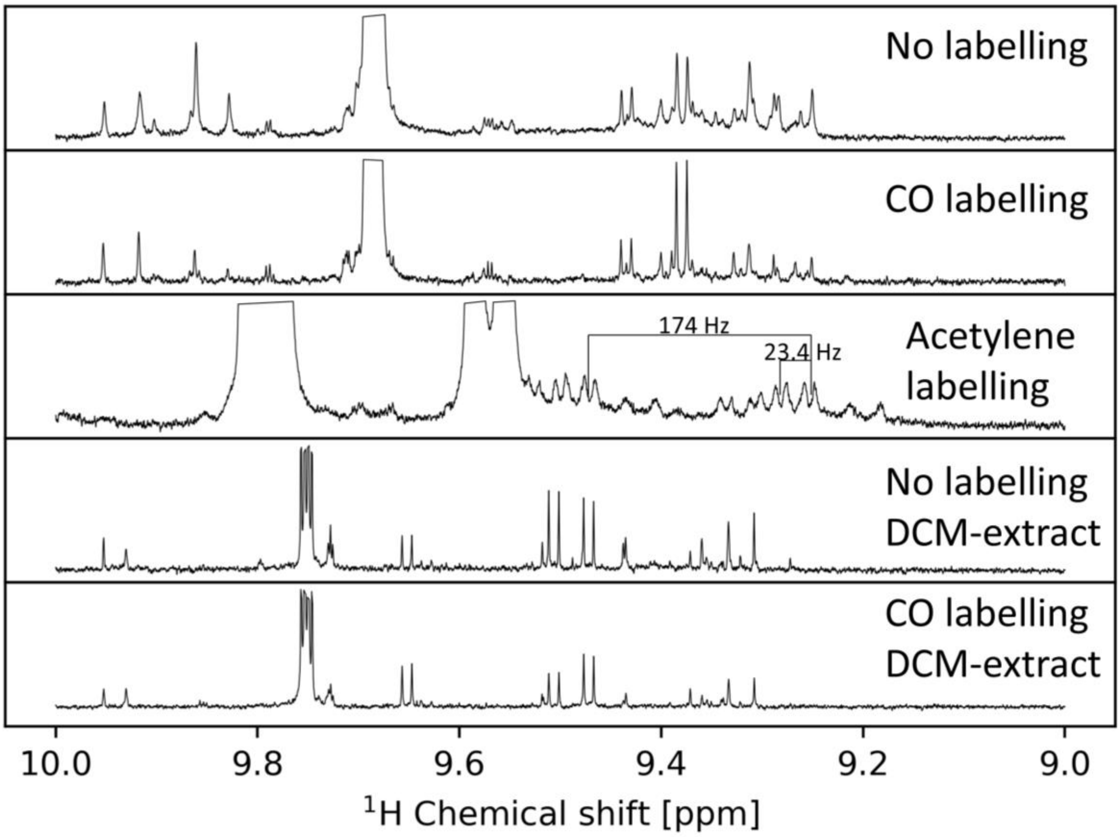

Supplementary Figure 3: 1D- ^{1}H NMR spectra of the aldehyde region for labelled and unlabelled samples. Additional couplings through ^{13}C labelling are added for the direct aldol condensation product of acetaldehyde, crotonaldehyde.

Acetaldehyde from acetylene alone

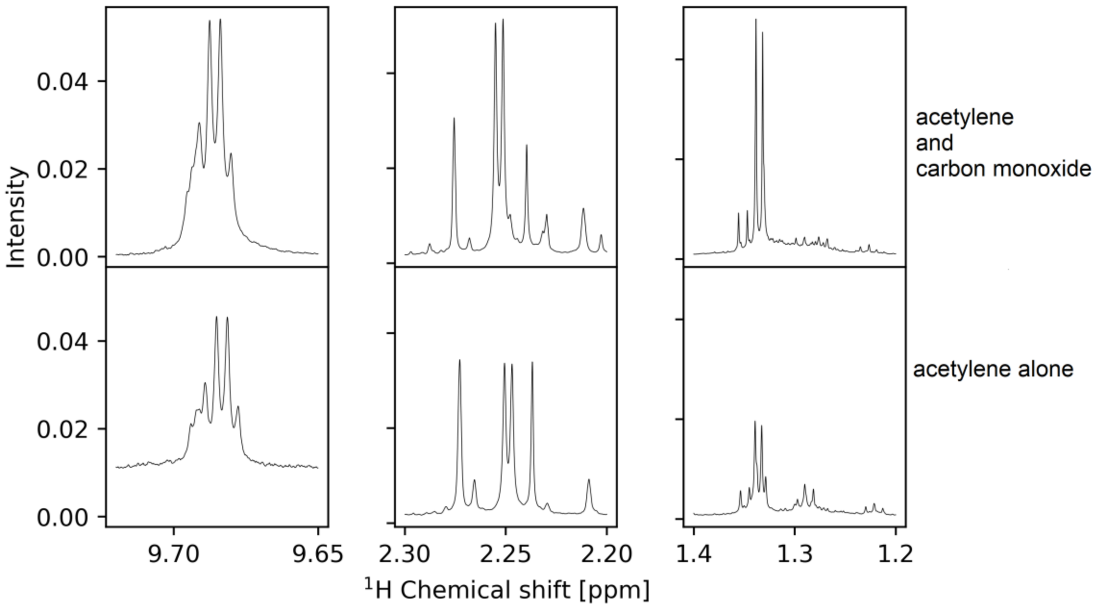

Supplementary Figure 4: Comparative acetaldehyde formation in setups with (top) and without (bottom) carbon monoxide. The NMR signals of the aldehyde hydrogen signal (column on the left), the methyl hydrogen of acetaldehyde (column in the middle), and the methyl hydrogen of the hydrate form of acetaldehyde (right column) are shown. The annotation on the right-hand side indicates the added catalyst, the incubation time, and the resulting yield in acetaldehyde.

Formic acid from acetylene

Based on the same principle, namely the splitting of the hydrogen signal, the origin of formic acid could be traced back partially to acetylene. Experiments with solely acetylene also produced formic acid.

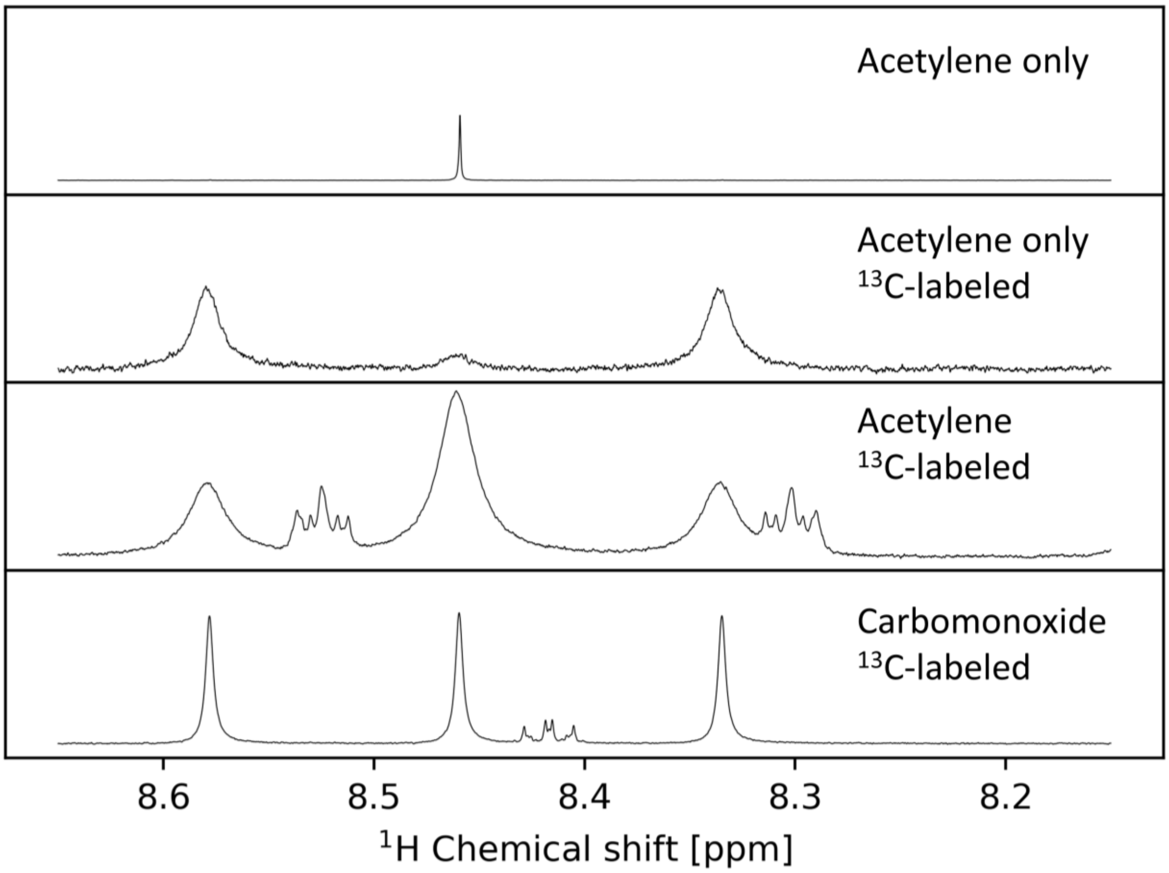

Supplementary Figure 5: 1D-1H NMR spectra of setups with varying ^{13}C labels showing the origin of formic acid.

pH influence on the reactivity of acetaldehyde

Prove of a strongly decreased aldol condensation of acetaldehyde under the exact reaction conditions. Acetaldehyde (300mM) was reacted in water containing NiS as a catalyst. The reaction time was 25 hours at 100°C. Acidification of the pH 4 reaction was achieved by adding formic acid to the water. The alkaline reaction also contained formic acid and was adjusted to pH 11 with sodium hydroxide. Both spectra are normalised to the TSP (0.2 mM) signal at 0 ppm. The acidified reaction only shows the first aldol condensation product but-2-enal. The alkaline reaction shows a much higher aldehyde diversity and greater complexity in general.

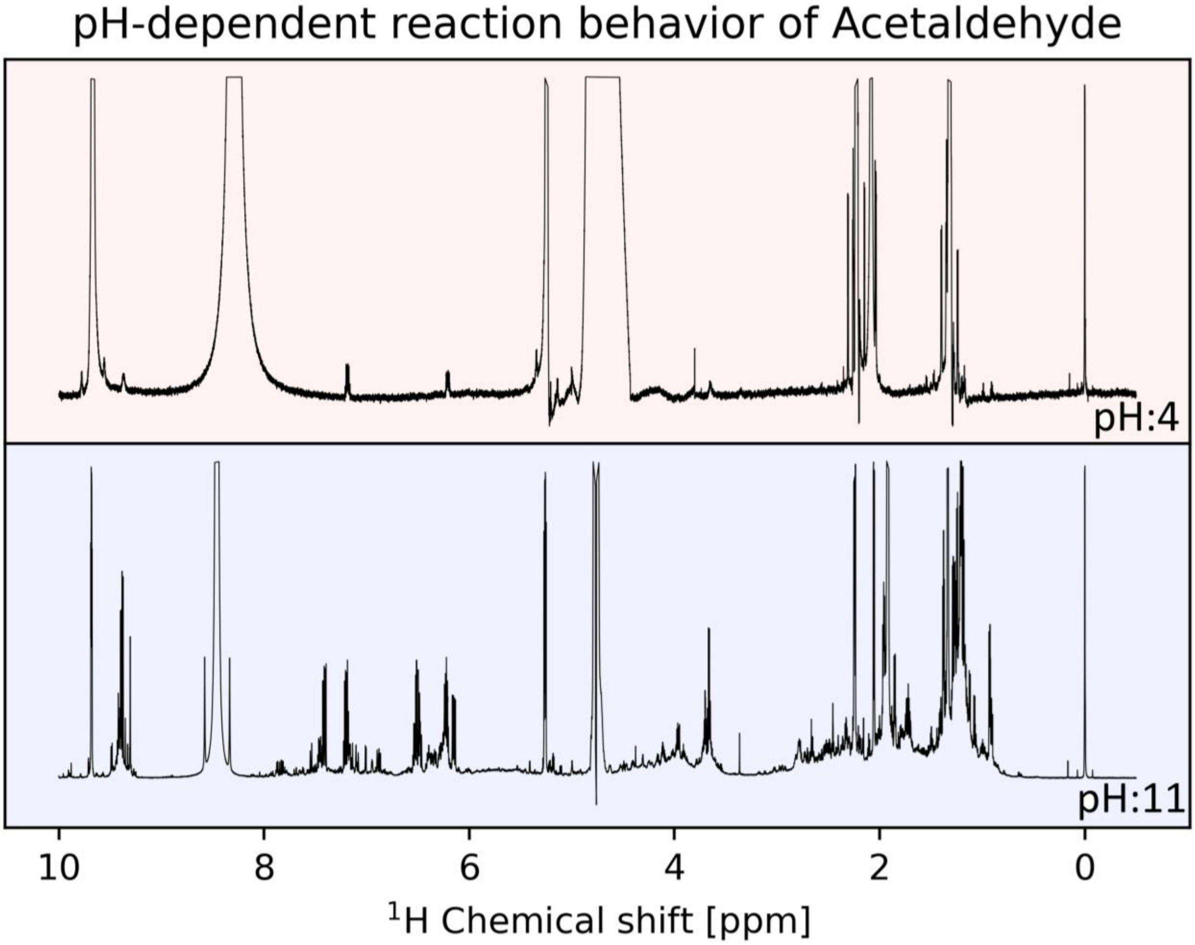

Supplementary Figure 6: 1D-1H NMR spectra of the same concentration of acetaldehyde standard in water over an NiS catalyst, spiked with formic acid at different pH. Samples were incubated for 24 hours at 378K.

Identification of 4-oxobutan-2-sulfonic acid

To prove the annotation of the ^{1}H-signals (9.69(dd), 3.55(ddd), 2.96(ddd), 2.72 (ddd), 1.35 (d)) with the structure of 4-oxobutan-2-sulfonic acid, a synthesis following the hypothesised formation route was performed. Acetaldehyde (5 mM) was reacted with a Na_2S-solution (10 mM in the reaction mixture) in water. After 60 minutes at 100°C, the annotated signals could be detected in a 1D-^{1}H spectrum. This reaction solution was then diluted in methanol and analysed at two different concentrations with an FT-ICR-MS showing a peak at m/z 151.00700 with an intensity correlating with the different concentration levels and not found in the blank. The high mass accuracy of FT-ICR-MS allowed for the direct annotation of the elemental composition, namely $C_4H_7O_4S^-$ as negative ionization mode was used for the detection of sulfonic acid. The exact elemental composition together with the connectivity and shift information of the NMR measurement made the identification of the compound possible.

Oxobutanesulfonic acid (hydrate): NMR (800 MHz, H2O/D2O): δ 5.22 (dd, J=4.95 Hz, 6.78 Hz, 1H), δ 3.02 (ddq, J=9.3 Hz, 4.64 Hz, 6.9 Hz, 1H), δ 2.17 (ddd, J =4.65 Hz, 6.9 Hz, 14 Hz, 1H), δ 1.75 (ddd, J = 4.96 Hz, 9.03 Hz, 14 Hz, 1H), 1.33 (d, J= 6.9 Hz, 3H), ^{13}C-^{1}H NMR (200MHz/800MHz, H2O/D2O): δ 92, 55, 42, 17.5.

Oxobutanesulfonic acid: NMR (800 MHz, H2O/D2O): δ 9.69 (dd, 1H), δ 3.54 (m, width=42 Hz, 1H), δ 2.96(ddd, J = 1.96 Hz, 6.45 Hz, 17.7 Hz, 1H), δ 2.72 (m, , width=40 Hz, 1H), 1.35 (d, J= 6.92 Hz, 3H), ^{13}C-^{1}H NMR (200MHz/800MHz, H2O/D2O): δ 207, 53, 43, 17; FT-ICR-MS (*m/z*): [M]- calcd. For $C_4H_7O_4S$, 151.00705; found, 151.00700.

Compound identification and quantification

The hydrogen and carbon shifts of all mentioned compounds are reported in the table below. The different methods of identification are noted in the column “Identification”. Standard means identification by spiking a standard into the mixture to observe an increase of the compound signal. For some compounds with very characteristic NMR shifts and high probability to be formed or unstable compounds only the NMR shifts are reported. Identification of 4-oxobutan-2-sulfonic acid was already described above. Signals used for quantification are also reported together with possible interferences leading to higher quantification error.

Compound	Identification	^{1}H-shift (ppm)	13C-shift (ppm)	Quantified signals	Interference
Acetaldehyde	Standard	9.68(q), 2.25(d)	33,<br>209.9	2.25 (d)	/
Acetaldehyde (hydrate)	Standard	5.26(q),<br>1.33(d)	26,<br>93	5.26(q)	Solvent suppression
Acrylic acid	Standard	6.14(dd),<br>6.03(dd),<br>5.66(dd)	125.5,<br>135.7,<br>177	6.14(dd), 6.03(dd),<br>5.66(dd)	/
Oxobutanesulfonic acid	1H/13C shift<br>LC-MS mass + fragmentation<br>FT-MS elemental composition	9.69(dd),<br>3.55(ddd),<br>2.96(ddd), 2.72 (ddd),<br>1.35 (d)	17,<br>43,<br>53,<br>207.6	/	/
Oxobutanesulfonic acid (hydrate)	1H/13C shift	5.22(dd),<br>3.02(m),<br>2.17(ddd), 1.75 (ddd),<br>1.33(d)	17.5,<br>42,<br>55,<br>92	5.22(dd)	Solvent suppression
Crotonaldehyde	1H/13C shift	9.38,<br>7.2,	201.69,	9.38(d)	/

		6.23,<br>2.05			
Alanine	Standard	1.25-1.48 (d),<br>3.6-3.8(q)<br>strong pH<br>dependency	23,<br>53,<br>178	1.48 (d)	Slight overlap
Lactic acid	Standard	4.13(q),<br>1.33(d)	/	4.13	/
Aminoethanol	1H/13C shift	1.194,<br>3.758	22.0,<br>66.8	1.194	/
Formic acid	Standard	8.46(s)	174	8.46	/
Acetic acid	1H/13C shift	1.97(s)	26,<br>184	1.97	/
Ethanol	1H shift	1.19(t),<br>3.66(q)	/	3.66	low S/N
Hydroxypropannitrile	1H/13C shift	1.569(d),<br>4.75(q)	23,<br>59,<br>123	1.57	/

Bottle setup	pH	Incubation time	Acetaldehyde (mM)	Acetaldehyde (hydrate) (mM)	Acrylic acid (mM)	Croton- aldehyde (mM)
S1	11	8 hours	0.16/0.48/0.45	0.24/0.91/1.10	0.46/0.28/0.20	nd
S1	10	24 hours	0.85/0.99/0.99	1.51/1.65/1.72	0.75/0.73/0.72	nd
S1	8	48 hours	2.07/1.03/1.14	3.07/1.60/1.66	0.766/0.88/0.93	0.04/qualitative
S1	7.6	7 days	1.08/1.01/1.22	1.24/1.19/1.32	0.60/0.57/0.46	qualitative
S1*	3	7days	18/19/18	19/22/21	nd	nd
S2	8	7 days	0.33/0.30/0.32	0.41/0.57/0.58	2.24/0.97/1.01	nd
S2*	3	7 days	11/13/13	13/16/17	nd	nd
**Bottle**	**pH**	**Incubation**	**Lactic acid (mM)**	**Aminoethanol (mM)**	**Formic acid (mM)**	**Acetic acid (mM)**
S1	11	8 hours	nd	nd	4.55/2.81/2.92	0.948/0.536/0.6
S1	10	24 hours	nd	nd	8.75/8.52/8.28	0.72/0.64/0.72
S1	8	48 hours	nd	nd	7.20/10.72/8.98	0.92/0.79/0.79
S1	7.6	7 days	nd	nd	8.48/9.36/11.7	1.02/0.98/1.032
S1*	3	7days	nd	nd	Not quantified	2.07/1.89
S2	8	7 days	nd	nd	3.17/2.00/2.01	0.50/0.33/0.31
S2*	3	7 days	0.16/0.17/0.17	nd	Not quantified	1.03/0.95
**Bottle**	**pH**	**Incubation**	**Alanine (mM)**	**Ethanol (mM)**	**2-hydroxypropannitrile (mM)**	**Oxobutanesulfonic acid (hydrate) (mM)**
S1	11	8 hours	nd	qualitative	nd	0.16/0.39/0.33
S1	10	24 hours	nd	qualitative	nd	0.64/0.62/0.70
S1	8	48 hours	nd	qualitative	nd	1.15/0.46/0.68
S1	7.6	7 days	nd	0.02/0.01/0.01	nd	0.6/0.57/0.58
S1*	3	7days	nd	0.59/0.43	nd	nd
S2	8	7 days	0.18/0.29/0.28	qualitative	nd	qualitative
S2*	3	7 days	nd	0.265/0.288	14.71/8.14/8.02	nd

Supplementary table 1: Concentration values of reported compounds for every replicate of the various setups. “nd” for not detected.

NMR experiment parameters for identification

Setup	Experiment	Spectral width (F2)	Spectral width (F1)	Number of scans	Increments	Relaxation delay (s)	Acquisition time (s)	Mixing time (ms)	1J coupling
S1	TOCSY	12 ppm	12 ppm	8	857	1.5	1	70	/
S1	HSQC	14 ppm	245 ppm	800	249	1.75	0.25	/	145
S2	TOCSY	11 ppm	11 ppm	16	1024	0.5	1.5	80	/
S2	HSQC-TOCSY	10 ppm	190 ppm	320	438	1.5	0.25	70	145
S2	HMBC	11 ppm	240 ppm	1280	137	0.5	1.5	/	145
S2*	HSQC	12 ppm	190 ppm	24	59	1.75	0.25	/	145
S2*	HMBC	12 ppm	230 ppm	256	39	0.5	1	/	145

Supplementary table 2: NMR experiment parameters for various spectra

LC-MS parameters

Materials

L-alanine (99%), acetonitrile and methanol (both LC-MS grade) were purchased from Merck (Darmstadt, Germany). Formic acid (98%, for mass spectrometry) was obtained from Honeywell Fluka (North Carolina, USA). Ammonium formate (10 M in water) was ordered from Sigma Aldrich (Steinheim, Germany). Purified water (18.2 MΩ) was from a Milli-Q integral system (Billerica, MA, USA). ESI-L low concentration tuning mix was supplied by Agilent (Santa Clara, CA, USA).

HILIC-MS/MS analysis

Samples were diluted 1:5 (*v*/*v*) with methanol, and analyzed by UPLC system (Waters Acquity, Milford, MA, USA) coupled to a Quadrupole time-of-flight (QTOF) mass spectrometer (MS) (Bruker maXis, Bremen, Germany). A hydrophilic interaction liquid chromatography (HILIC) column ZIC-cHILIC (100 x 2.1 mm, 3 µm, Merck, Darmstadt, Germany) was used at flow rate of 0.5 mL/min under 40 °C for chromatographic separation. 5:95 (*v*/*v*) acetonitrile:water and 95:5 (*v*/*v*) acetonitrile:water, both with 5 mM ammonium formate and 0.1% formic acid, were used as eluent A and B, respectively. The gradient was: 0 min, 99.9% B; 2 min, 99.9% B; 13 min, 56% B; 14 min, 30% B; 14.1 min, 10 %B; 16 min, 10 %B; 16.1 min, 99.9 %B. The column was equilibrated at 99.9 %B for 3 min after each injection and 5 µL was injected for each sample.

The MS was operated in both positive and negative ionization mode with mass range from m/z 50 to m/z 1500. MS settings were: nebulizer pressure 2 bar, capillary voltage 4500 V for positive mode and 4000 V for negative mode, dry gas 10 L/min with temperature 200 °C. Data were obtained with an acquisition rate of 5 Hz in data-dependent mode. MS/MS spectra were triggered for the three highest MS1 ions in each precursor scan with collision energy of 20 eV. Diluted ESI-L tuning mix (1:4 (*v*/*v*) with 75:25 (*v*/*v*) acetonitrile:water) was analyzed from 0.1 to 0.3 minutes in each measurement for internal recalibration.

Data processing

Raw HILIC-MS/MS data were calibrated and converted to mzXML format by Bruker DataAnalysis 5.0 software (Bremen, Germany). The data processing, including peak

picking, peak alignment and correspondence, feature annotation, and data cleaning were done using in-house script based on XCMS and CAMERA packages in R[1, 2]. The fragmentation pattern, characterized by fragment ions of HSO_3^- (*m/z* 80.9641), $SO_3^{\bullet-}$ radicals (*m/z* 79.9573), $HSO4^-$ (*m/z* 96.9590), and neutral losses of H_2SO_3(81.9725 Da), SO_3(79.9568 Da), H_2SO_4(97.9674 Da) for sulfonic acids in negative ionization mode, were summarized and used for searching compounds containing the sulfonic acid group[3, 4]. The consensus MS/MS spectra were screened with an error of 0.005 Da and features with MS/MS spectra containing at least $HSO3^-$ fragments were kept as candidates. The formula of potential sulfonic acids was calculated by SIRIUS software based on MS/MS spectra [5]. The calculated formula with explained intensity > 0.6 and absolute ppm error < 10 were kept.

Alanine identified in the alkaline setup by LC-MS/MS							
**Metabolite Name**	**Molecular formula**	**RT**	**m/z**	**Theoretical m/z[1]**	**Error (ppm)**	**Ionization mode**	**Confidence Level[2]**
Alanine	C3H7O2N	8,24	90,0544	90,0550	6,10	positive	Level 2
Alanine	C3H7O2N	8,31	88,0395	88,0393	2,21	negative	Level 2
1: The theoretical m/z was calculated as $[M+H]^+$ for positive mode and $[M-H]^-$ for negative mode<br>2: The amino acids were identified by comparing to the reference standards analyzed with the same LC-MS/MS method. The identification level was assigned following the Metabolomics Standards Initiative [1]:<br>Level 1: retention time difference < 0.2 min, error of m/z < 10 ppm, MS/MS spectra (main fragment ions matching within 0.01 Da)<br>Level 2: retention time difference < 0.2 min, error of m/z < 10 ppm							
[1] Sumner L W, Amberg A, Barrett D, et al. Proposed minimum reporting standards for chemical analysis[J]. Metabolomics, 2007, 3(3): 211-221.							

Supplementtray table 3 Identification of alanine via LC-MS/MS

Molecular formulas showing fragment characteristic for sulfonic acids

m/z	Retention time (min.)	Molecular formula	Adduct	Fragment HSO3-	Fragment SO3-	Neutral loss H2SO3	Neutral loss SO3	Theoretical m/z	Error (ppm)
106,9799	4,95	C2H4O3S	[M - H]-	Yes	Yes	NO	NO	106,9797	1,48
126,9515	3,91	CH4O3S2	[M - H]-	Yes	Yes	NO	NO	126,9518	-2,46
138,9519	0,58	C2H4O3S2	[M - H]-	Yes	Yes	NO	NO	138,9518	0,63
139,006	5,57	C3H8O4S	[M - H]-	Yes	Yes	NO	NO	139,0060	0,32
140,9671	3,45	C2H6O3S2	[M - H]-	Yes	Yes	NO	Yes	140,9675	-2,57
150,9697	8,72	C3H4O5S	[M - H]-	Yes	Yes	NO	NO	150,9696	0,86
151,006	4,97	C4H8O4S	[M - H]-	Yes	Yes	NO	NO	151,0060	0,29
151,0061	4,40	C4H8O4S	[M - H]-	Yes	Yes	NO	NO	151,0060	0,95
152,9859	8,12	C3H6O5S	[M - H]-	Yes	Yes	Yes	NO	152,9852	4,44
154,9468	0,94	C2H4O4S2	[M - H]-	Yes	Yes	NO	NO	154,9467	0,47
154,9837	3,14	C3H8O3S2	[M - H]-	Yes	Yes	NO	Yes	154,9831	3,79
161,0271	4,35	C6H10O3S	[M - H]-	Yes	Yes	NO	NO	161,0267	2,54
165,0217	4,72	C5H10O4S	[M - H]-	Yes	Yes	NO	NO	165,0216	0,57
166,9473	1,29	C3H4O4S2	[M - H]-	Yes	NO	NO	Yes	166,9467	3,44
166,983	4,26	C4H8O3S2	[M - H]-	Yes	Yes	NO	NO	166,9831	-0,67
166,9831	12,82	C4H8O3S2	[M - H]-	Yes	Yes	NO	NO	166,9831	-0,07
167,0009	4,64	C4H8O5S	[M - H]-	Yes	Yes	NO	NO	167,0009	0,18

167,0011	7,89	C4H8O5S	[M - H]-	Yes	Yes	Yes	NO	167,0009	1,37
168,9988	2,87	C4H10O3S2	[M - H]-	Yes	Yes	NO	NO	168,9988	0,22
170,9238	6,89	C2H4O3S3	[M - H]-	Yes	Yes	NO	NO	170,9239	-0,48
170,9415	8,30	C2H4O5S2	[M - H]-	Yes	NO	Yes	Yes	170,9416	-0,83
170,9421	10,25	C2H4O5S2	[M - H]-	Yes	Yes	NO	NO	170,9416	2,68
174,919	2,51	CH4O4S3	[M - H]-	Yes	Yes	NO	NO	174,9188	1,16
175,0057	5,12	C6H8O4S	[M - H]-	Yes	Yes	NO	NO	175,0060	-1,46
177,0218	5,28	C6H10O4S	[M - H]-	Yes	Yes	NO	NO	177,0216	1,10
179,0373	4,53	C6H12O4S	[M - H]-	Yes	Yes	NO	NO	179,0373	0,25
180,9799	6,16	C4H6O6S	[M - H]-	Yes	Yes	Yes	Yes	180,9801	-1,30
180,9801	10,00	C4H6O6S	[M - H]-	Yes	Yes	NO	NO	180,9801	-0,19
180,9801	9,77	C4H6O6S	[M - H]-	Yes	Yes	NO	NO	180,9801	-0,19
181,0168	5,67	C5H10O5S	[M - H]-	Yes	Yes	NO	NO	181,0165	1,54
184,9572	7,24	C3H6O5S2	[M - H]-	Yes	NO	NO	Yes	184,9573	-0,49
188,9517	9,91	C2H6O6S2	[M - H]-	Yes	Yes	Yes	NO	188,9522	-2,68
189,0212	5,02	C7H10O4S	[M - H]-	Yes	Yes	Yes	NO	189,0216	-2,15
190,8959	1,92	CH4O3S4	[M - H]-	Yes	Yes	NO	Yes	190,8960	-0,28
190,9137	9,81	CH4O5S3	[M - H]-	Yes	NO	NO	NO	190,9137	-0,06
191,0002	5,34	C6H8O5S	[M - H]-	Yes	Yes	NO	NO	191,0009	-3,51
191,0013	8,04	C6H8O5S	[M - H]-	Yes	Yes	NO	NO	191,0009	2,25

191,0374	4,36	C7H12O4S	[M - H]-	Yes	Yes	NO	NO	191,0373	0,75
192,9986	4,21	C6H10O3S2	[M - H]-	Yes	Yes	NO	NO	192,9988	-0,84
193,0164	4,34	C6H10O5S	[M - H]-	Yes	Yes	NO	NO	193,0165	-0,62
193,0171	8,03	C6H10O5S	[M - H]-	Yes	Yes	NO	NO	193,0165	3,00
194,924	9,18	C4H4O3S3	[M - H]-	Yes	Yes	NO	Yes	194,9239	0,60
194,9415	5,72	C4H4O5S2	[M - H]-	Yes	Yes	NO	NO	194,9416	-0,72
194,9956	8,05	C5H8O6S	[M - H]-	Yes	NO	NO	NO	194,9958	-0,95
194,9959	9,64	C5H8O6S	[M - H]-	Yes	Yes	Yes	NO	194,9958	0,59
195,0319	6,04	C6H12O5S	[M - H]-	Yes	Yes	NO	NO	195,0322	-1,39
195,0323	5,49	C6H12O5S	[M - H]-	Yes	Yes	NO	NO	195,0322	0,66
195,0324	3,24	C6H12O5S	[M - H]-	Yes	Yes	NO	NO	195,0322	1,18
196,9394	4,00	C4H6O3S3	[M - H]-	Yes	Yes	NO	Yes	196,9395	-0,67
196,9567	3,50	C4H6O5S2	[M - H]-	Yes	NO	NO	NO	196,9573	-3,00
196,9751	9,69	C4H6O7S	[M - H]-	Yes	Yes	NO	NO	196,9750	0,26
197,0117	8,40	C5H10O6S	[M - H]-	Yes	Yes	Yes	NO	197,0114	1,34
197,0477	6,97	C6H14O5S	[M - H]-	Yes	Yes	NO	NO	197,0478	-0,61
198,0179	8,46	C5H11O6S	[M - H]-	Yes	Yes	NO	NO	198,0193	-6,87
198,9552	2,01	C4H8O3S3	[M - H]-	Yes	Yes	NO	Yes	198,9552	0,09
198,9729	5,42	C4H8O5S2	[M - H]-	Yes	NO	Yes	Yes	198,9729	-0,21
198,9731	3,03	C4H8O5S2	[M - H]-	Yes	Yes	NO	NO	198,9729	0,80

198,9731	2,60	C4H8O5S2	[M - H]-	Yes	Yes	NO	NO	198,9729	0,80
200,88	10,57	C2H2O3S4	[M - H]-	Yes	Yes	NO	NO	200,8803	-1,51
200,8804	11,56	C2H2O3S4	[M - H]-	Yes	Yes	NO	NO	200,8803	0,48
201,0217	4,37	C8H10O4S	[M - H]-	Yes	NO	NO	NO	201,0216	0,47
202,8962	4,54	C2H4O3S4	[M - H]-	Yes	Yes	NO	NO	202,8960	1,22
202,9136	8,81	C2H4O5S3	[M - H]-	Yes	Yes	NO	Yes	202,9137	-0,55
202,9136	13,21	C2H4O5S3	[M - H]-	Yes	Yes	NO	NO	202,9137	-0,55
202,9136	7,01	C2H4O5S3	[M - H]-	Yes	Yes	NO	Yes	202,9137	-0,55
203,0366	4,63	C8H12O4S	[M - H]-	Yes	Yes	NO	NO	203,0373	-3,23
204,9113	1,63	CH2O8S2	[M - H]-	Yes	Yes	NO	NO	204,9107	2,76
204,9299	9,43	C2H6O5S3	[M - H]-	Yes	Yes	NO	NO	204,9294	2,63
205,0165	8,40	C7H10O5S	[M - H]-	Yes	NO	NO	NO	205,0165	-0,10
205,0166	4,02	C7H10O5S	[M - H]-	Yes	Yes	NO	NO	205,0165	0,39
205,0167	3,78	C7H10O5S	[M - H]-	Yes	NO	NO	NO	205,0165	0,87
205,0533	4,16	C8H14O4S	[M - H]-	Yes	Yes	NO	NO	205,0529	1,92
206,9957	9,66	C6H8O6S	[M - H]-	Yes	Yes	Yes	NO	206,9958	-0,41
206,9958	5,12	C6H8O6S	[M - H]-	Yes	Yes	NO	NO	206,9958	0,07
207,032	3,28	C7H12O5S	[M - H]-	Yes	NO	NO	NO	207,0322	-0,82
207,0321	5,85	C7H12O5S	[M - H]-	Yes	Yes	Yes	NO	207,0322	-0,34
209,0475	5,41	C7H14O5S	[M - H]-	Yes	NO	NO	NO	209,0478	-1,53

209,0476	5,17	C7H14O5S	[M - H]-	Yes	NO	NO	NO	209,0478	-1,06
210,9723	2,72	C5H8O5S2	[M - H]-	Yes	NO	NO	NO	210,9729	-3,04
210,9729	3,04	C5H8O5S2	[M - H]-	Yes	Yes	NO	Yes	210,9729	-0,20
210,9731	7,23	C5H8O5S2	[M - H]-	Yes	Yes	NO	NO	210,9729	0,75
210,9895	4,02	C5H8O7S	[M - H]-	Yes	NO	NO	NO	210,9907	-5,69
211,0094	4,71	C6H12O4S2	[M - H]-	Yes	Yes	NO	NO	211,0093	0,35
212,9854	12,03	C8H6O5S	[M - H]-	Yes	NO	NO	NO	212,9852	0,84
212,9882	10,12	C5H10O5S2	[M - H]-	Yes	Yes	NO	NO	212,9886	-1,84
212,9883	7,59	C5H10O5S2	[M - H]-	Yes	NO	NO	NO	212,9886	-1,37
215,0018	11,64	C8H8O5S	[M - H]-	Yes	NO	NO	NO	215,0009	4,32
216,9296	5,13	C3H6O5S3	[M - H]-	Yes	Yes	NO	Yes	216,9294	1,10
217,0528	4,45	C9H14O4S	[M - H]-	Yes	Yes	NO	NO	217,0529	-0,49
219,0141	4,31	C8H12O3S2	[M - H]-	Yes	Yes	NO	NO	219,0144	-1,43
219,0325	5,63	C8H12O5S	[M - H]-	Yes	NO	NO	NO	219,0322	1,50
220,924	8,98	CH2O11S	[M - H]-	Yes	Yes	NO	Yes	220,9234	2,68
221,0116	4,74	C7H10O6S	[M - H]-	Yes	Yes	NO	Yes	221,0114	0,75
221,0473	4,79	C8H14O5S	[M - H]-	Yes	Yes	NO	NO	221,0478	-2,36
221,0475	4,16	C8H14O5S	[M - H]-	Yes	Yes	NO	NO	221,0478	-1,45
221,0482	5,15	C8H14O5S	[M - H]-	Yes	Yes	NO	NO	221,0478	1,72
223,0267	5,92	C7H12O6S	[M - H]-	Yes	Yes	NO	Yes	223,0271	-1,73

223,0267	5,34	C7H12O6S	[M - H]-	Yes	NO	NO	NO	223,0271	-1,73
223,0269	6,41	C7H12O6S	[M - H]-	Yes	NO	NO	NO	223,0271	-0,83
223,0273	4,49	C7H12O6S	[M - H]-	Yes	Yes	NO	NO	223,0271	0,96
223,0628	5,22	C8H16O5S	[M - H]-	Yes	Yes	NO	NO	223,0635	-3,01
226,8959	2,04	C4H4O3S4	[M - H]-	Yes	Yes	NO	Yes	226,8960	-0,23
226,9136	9,56	C4H4O5S3	[M - H]-	Yes	NO	NO	NO	226,9137	-0,49
226,9311	10,14	C4H4O7S2	[M - H]-	Yes	NO	NO	Yes	226,9315	-1,63
227,0044	6,60	C6H12O5S2	[M - H]-	Yes	NO	NO	NO	227,0042	0,70
227,0219	8,37	C6H12O7S	[M - H]-	Yes	NO	NO	NO	227,0220	-0,44
228,9112	12,65	C4H6O3S4	[M - H]-	Yes	Yes	NO	NO	228,9116	-1,76
228,9112	12,00	C4H6O3S4	[M - H]-	Yes	Yes	NO	NO	228,9116	-1,76
228,9288	5,43	C4H6O5S3	[M - H]-	Yes	NO	NO	NO	228,9294	-2,45
228,9833	6,39	C5H10O6S2	[M - H]-	Yes	NO	NO	Yes	228,9835	-0,90
230,9447	4,79	C4H8O5S3	[M - H]-	Yes	Yes	NO	NO	230,9450	-1,35
231,0325	3,06	C9H12O5S	[M - H]-	Yes	Yes	NO	NO	231,0322	1,43
231,0686	4,10	C10H16O4S	[M - H]-	Yes	Yes	NO	NO	231,0686	0,19
233,0479	5,36	C9H14O5S	[M - H]-	Yes	Yes	NO	NO	233,0478	0,34
234,8682	3,12	C2H4O3S5	[M - H]-	Yes	NO	NO	NO	234,8680	0,75
234,8852	8,13	C2H4O5S4	[M - H]-	Yes	Yes	NO	Yes	234,8858	-2,48
234,8854	7,02	C2H4O5S4	[M - H]-	Yes	Yes	NO	Yes	234,8858	-1,63

234,9902	9,59	C7H8O7S	[M - H]-	Yes	Yes	Yes	NO	234,9907	-2,13
235,0093	4,61	C8H12O4S2	[M - H]-	Yes	Yes	NO	NO	235,0093	-0,11
235,0095	4,96	C8H12O4S2	[M - H]-	Yes	Yes	NO	NO	235,0093	0,74
235,0268	9,50	C8H12O6S	[M - H]-	Yes	Yes	NO	NO	235,0271	-1,21
235,0269	4,57	C8H12O6S	[M - H]-	Yes	Yes	NO	Yes	235,0271	-0,79
235,0271	6,21	C8H12O6S	[M - H]-	Yes	NO	NO	NO	235,0271	0,06
235,0271	6,02	C8H12O6S	[M - H]-	Yes	NO	NO	NO	235,0271	0,06
236,9874	3,04	C7H10O5S2	[M - H]-	Yes	NO	NO	Yes	236,9886	-5,03
237,0426	6,40	C8H14O6S	[M - H]-	Yes	Yes	Yes	NO	237,0427	-0,57
238,9498	4,16	C6H8O4S3	[M - H]-	Yes	Yes	Yes	Yes	238,9501	-1,24
239,0042	6,06	C7H12O5S2	[M - H]-	Yes	Yes	NO	NO	239,0042	-0,17
239,0209	6,57	C7H12O7S	[M - H]-	Yes	Yes	NO	NO	239,0220	-4,60
239,0582	5,13	C8H16O6S	[M - H]-	Yes	NO	NO	NO	239,0584	-0,78
239,0583	5,46	C8H16O6S	[M - H]-	Yes	Yes	NO	NO	239,0584	-0,36
242,9451	5,70	C5H8O5S3	[M - H]-	Yes	Yes	NO	Yes	242,9450	0,36
242,9814	3,76	C6H12O4S3	[M - H]-	Yes	Yes	NO	Yes	242,9814	0,01
244,9604	4,61	C5H10O5S3	[M - H]-	Yes	Yes	NO	NO	244,9607	-1,07
244,9606	6,11	C5H10O5S3	[M - H]-	Yes	NO	NO	NO	244,9607	-0,25
246,9398	9,14	C4H8O6S3	[M - H]-	Yes	Yes	Yes	NO	246,9399	-0,51
247,0266	8,09	C9H12O6S	[M - H]-	Yes	NO	NO	NO	247,0271	-1,96

247,0636	5,20	C10H16O5S	[M - H]-	Yes	NO	NO	NO	247,0635	0,52
247,0641	4,82	C10H16O5S	[M - H]-	Yes	Yes	NO	NO	247,0635	2,55
248,9016	4,72	C3H6O5S4	[M - H]-	Yes	Yes	NO	Yes	248,9014	0,67
249,0426	6,47	C9H14O6S	[M - H]-	Yes	Yes	Yes	NO	249,0427	-0,54
249,0426	5,87	C9H14O6S	[M - H]-	Yes	NO	Yes	NO	249,0427	-0,54
249,043	9,28	C9H14O6S	[M - H]-	Yes	Yes	NO	NO	249,0427	1,06
250,8806	6,95	C2H4O6S4	[M - H]-	Yes	Yes	NO	Yes	250,8807	-0,39
251,0582	5,47	C9H16O6S	[M - H]-	Yes	NO	Yes	NO	251,0584	-0,74
251,0585	6,13	C9H16O6S	[M - H]-	Yes	Yes	Yes	NO	251,0584	0,46
252,9115	1,66	C6H6O3S4	[M - H]-	Yes	NO	NO	Yes	252,9116	-0,41
252,9653	4,23	C7H10O4S3	[M - H]-	Yes	Yes	NO	Yes	252,9657	-1,77
253,0012	8,90	C7H10O8S	[M - H]-	Yes	Yes	NO	NO	253,0013	-0,25
253,0016	8,48	C7H10O8S	[M - H]-	Yes	NO	NO	Yes	253,0013	1,33
254,9991	6,13	C7H12O6S2	[M - H]-	Yes	NO	NO	NO	254,9992	-0,22
255,0168	8,53	C7H12O8S	[M - H]-	Yes	NO	NO	NO	255,0169	-0,45
256,9421	3,96	C5H6O8S2	[M - H]-	Yes	Yes	NO	NO	256,9420	0,25
256,9778	8,65	C6H10O7S2	[M - H]-	Yes	Yes	NO	NO	256,9784	-2,41
256,9786	8,90	C6H10O7S2	[M - H]-	Yes	Yes	Yes	NO	256,9784	0,70
258,9939	4,97	C6H12O7S2	[M - H]-	Yes	Yes	NO	NO	258,9941	-0,66
258,9942	9,37	C6H12O7S2	[M - H]-	Yes	Yes	Yes	NO	258,9941	0,50

259,0632	4,83	C11H16O5S	[M - H]-	Yes	Yes	NO	NO	259,0635	-1,05
260,9009	1,27	C4H6O5S4	[M - H]-	Yes	Yes	NO	Yes	260,9014	-2,04
260,9019	2,11	C4H6O5S4	[M - H]-	Yes	Yes	NO	NO	260,9014	1,79
260,9734	10,73	C5H10O8S2	[M - H]-	Yes	NO	Yes	NO	260,9733	0,25
261,0095	10,10	C6H14O7S2	[M - H]-	Yes	NO	NO	NO	261,0097	-0,85
261,025	2,66	C10H14O4S2	[M - H]-	Yes	NO	NO	NO	261,0250	0,09
261,0251	4,05	C10H14O4S2	[M - H]-	Yes	Yes	NO	NO	261,0250	0,47
261,0421	7,04	C10H14O6S	[M - H]-	Yes	Yes	NO	NO	261,0427	-2,43
261,0428	5,86	C10H14O6S	[M - H]-	Yes	NO	Yes	NO	261,0427	0,25
261,0786	4,64	C11H18O5S	[M - H]-	Yes	Yes	NO	NO	261,0791	-2,00
263,0217	8,84	C9H12O7S	[M - H]-	Yes	NO	Yes	NO	263,0220	-1,14
263,0404	4,29	C10H16O4S2	[M - H]-	Yes	Yes	Yes	NO	263,0406	-0,86
263,041	2,30	C10H16O4S2	[M - H]-	Yes	NO	NO	NO	263,0406	1,42
264,9836	4,50	C8H10O6S2	[M - H]-	Yes	Yes	NO	Yes	264,9835	0,35
265,0015	9,26	C8H10O8S	[M - H]-	Yes	Yes	Yes	NO	265,0013	0,89
265,0197	6,29	C9H14O5S2	[M - H]-	Yes	NO	NO	NO	265,0199	-0,72
265,0371	6,20	C9H14O7S	[M - H]-	Yes	NO	NO	NO	265,0377	-2,08
265,0371	5,33	C9H14O7S	[M - H]-	Yes	NO	NO	NO	265,0377	-2,08
266,9805	4,13	C8H12O4S3	[M - H]-	Yes	Yes	NO	Yes	266,9814	-3,36
268,9071	3,66	C6H6O4S4	[M - H]-	Yes	Yes	NO	Yes	268,9065	2,16

268,9782	8,74	C7H10O7S2	[M - H]-	Yes	Yes	Yes	Yes	268,9784	-0,82
268,9786	9,37	C7H10O7S2	[M - H]-	Yes	Yes	Yes	Yes	268,9784	0,67
269,0324	9,21	C8H14O8S	[M - H]-	Yes	Yes	NO	NO	269,0326	-0,61
270,976	5,34	C7H12O5S3	[M - H]-	Yes	NO	NO	NO	270,9763	-1,15
270,976	3,72	C7H12O5S3	[M - H]-	Yes	NO	NO	NO	270,9763	-1,15
270,9938	9,47	C7H12O7S2	[M - H]-	Yes	Yes	Yes	NO	270,9941	-1,00
270,9941	8,11	C7H12O7S2	[M - H]-	Yes	NO	NO	NO	270,9941	0,11
273,0097	9,29	C7H14O7S2	[M - H]-	Yes	NO	Yes	NO	273,0097	-0,08
274,9712	4,78	C6H12O6S3	[M - H]-	Yes	NO	NO	NO	274,9712	-0,10
274,9888	10,57	C6H12O8S2	[M - H]-	Yes	NO	NO	NO	274,9890	-0,67
275,0583	3,01	C11H16O6S	[M - H]-	Yes	NO	Yes	NO	275,0584	-0,31
275,0589	5,49	C11H16O6S	[M - H]-	Yes	NO	NO	NO	275,0584	1,87
277,0202	4,86	C10H14O5S2	[M - H]-	Yes	NO	NO	NO	277,0199	1,11
277,0733	5,72	C11H18O6S	[M - H]-	Yes	NO	NO	NO	277,0740	-2,65
277,0736	5,33	C11H18O6S	[M - H]-	Yes	NO	Yes	NO	277,0740	-1,57
277,0738	6,09	C11H18O6S	[M - H]-	Yes	NO	NO	NO	277,0740	-0,85
278,9999	4,86	C9H12O6S2	[M - H]-	Yes	NO	NO	NO	278,9992	2,67
280,9429	3,85	C7H6O8S2	[M - H]-	Yes	Yes	NO	NO	280,9420	3,08
282,8526	6,81	C2H4O6S5	[M - H]-	Yes	Yes	NO	NO	282,8528	-0,59
282,9761	4,77	C8H12O5S3	[M - H]-	Yes	Yes	NO	NO	282,9763	-0,75

285,0093	9,70	C8H14O7S2	[M - H]-	Yes	NO	NO	NO	285,0097	-1,48
285,0099	8,05	C8H14O7S2	[M - H]-	Yes	NO	NO	NO	285,0097	0,63
285,0427	5,43	C12H14O6S	[M - H]-	Yes	NO	NO	NO	285,0427	-0,12
287,0251	9,14	C8H16O7S2	[M - H]-	Yes	Yes	Yes	NO	287,0254	-0,94
287,0586	6,13	C12H16O6S	[M - H]-	Yes	NO	Yes	NO	287,0584	0,75
288,9674	12,17	C6H10O9S2	[M - H]-	Yes	NO	Yes	NO	288,9682	-2,94
288,9678	10,38	C6H10O9S2	[M - H]-	Yes	NO	NO	Yes	288,9682	-1,56
289,0557	4,74	C15H14O4S	[M - H]-	Yes	Yes	NO	NO	289,0529	9,67
290,9989	5,81	C10H12O6S2	[M - H]-	Yes	NO	Yes	NO	290,9992	-0,88
293,0132	6,53	C13H10O6S	[M - H]-	Yes	NO	NO	NO	293,0114	6,02
293,0141	5,92	C10H14O6S2	[M - H]-	Yes	NO	NO	NO	293,0148	-2,41
294,9424	1,34	C5H12O6S4	[M - H]-	Yes	Yes	NO	NO	294,9433	-3,04
296,9554	5,43	C8H10O6S3	[M - H]-	Yes	NO	NO	NO	296,9556	-0,59
297,0098	9,34	C9H14O7S2	[M - H]-	Yes	Yes	Yes	Yes	297,0097	0,27
297,0272	9,14	C9H14O9S	[M - H]-	Yes	NO	NO	NO	297,0275	-0,94
297,0798	4,47	C14H18O5S	[M - H]-	Yes	Yes	NO	NO	297,0791	2,29
303,0893	5,36	C13H20O6S	[M - H]-	Yes	NO	Yes	NO	303,0897	-1,27
304,945	11,60	C6H10O8S3	[M - H]-	Yes	NO	NO	NO	304,9454	-1,33
306,8523	7,10	C4H4O6S5	[M - H]-	Yes	NO	NO	Yes	306,8528	-1,52
306,9788	8,13	C6H12O10S2	[M - H]-	Yes	NO	NO	NO	306,9788	-0,05

308,8673	7,10	C4H6O6S5	[M - H]-	Yes	NO	NO	NO	308,8684	-3,62
308,868	6,81	C4H6O6S5	[M - H]-	Yes	NO	NO	Yes	308,8684	-1,35
313,0039	9,01	C9H14O8S2	[M - H]-	Yes	Yes	Yes	NO	313,0046	-2,35
313,0562	3,82	C14H18O4S2	[M - H]-	Yes	Yes	NO	NO	313,0563	-0,25
314,9836	12,16	C8H12O9S2	[M - H]-	Yes	NO	Yes	NO	314,9839	-0,95
315,0203	9,43	C9H16O8S2	[M - H]-	Yes	NO	Yes	NO	315,0203	0,05
316,9984	10,06	C8H14O9S2	[M - H]-	Yes	NO	Yes	NO	316,9995	-3,63
319,0304	5,73	C12H16O6S2	[M - H]-	Yes	NO	NO	NO	319,0305	-0,18
320,9761	10,15	C7H14O8S3	[M - H]-	Yes	NO	Yes	NO	320,9767	-1,89
322,9703	5,83	C10H12O6S3	[M - H]-	Yes	Yes	NO	NO	322,9712	-2,87
324,8627	10,25	C4H6O7S5	[M - H]-	Yes	NO	NO	NO	324,8633	-1,95
325,002	10,09	C13H10O8S	[M - H]-	Yes	Yes	Yes	NO	325,0013	2,26
326,8789	11,89	C3H4O12S3	[M - H]-	Yes	NO	NO	NO	326,8781	2,40
328,9991	10,65	C9H14O9S2	[M - H]-	Yes	NO	NO	Yes	328,9995	-1,37
329,0356	9,72	C13H14O8S	[M - H]-	Yes	NO	Yes	Yes	329,0326	9,23
331,0151	10,08	C9H16O9S2	[M - H]-	Yes	NO	Yes	NO	331,0152	-0,30
333,0456	5,90	C13H18O6S2	[M - H]-	Yes	NO	Yes	NO	333,0461	-1,52
333,0456	5,45	C13H18O6S2	[M - H]-	Yes	NO	Yes	NO	333,0461	-1,52
336,9862	5,08	C11H14O6S3	[M - H]-	Yes	NO	NO	NO	336,9869	-2,01
339,0896	5,41	C16H20O6S	[M - H]-	Yes	NO	NO	NO	339,0897	-0,25

341,036	5,03	C11H18O8S2	[M - H]-	Yes	NO	NO	NO	341,0359	0,19
344,9757	10,30	C9H14O8S3	[M - H]-	Yes	NO	NO	Yes	344,9767	-2,92
345,0306	9,84	C13H14O9S	[M - H]-	Yes	NO	Yes	NO	345,0275	9,04
346,9371	8,62	C7H8O12S2	[M - H]-	Yes	NO	NO	Yes	346,9373	-0,70
346,9374	8,29	C7H8O12S2	[M - H]-	Yes	NO	Yes	Yes	346,9373	0,16
347,0456	10,25	C13H16O9S	[M - H]-	Yes	NO	Yes	NO	347,0431	7,12
357,0305	9,68	C11H18O9S2	[M - H]-	Yes	NO	Yes	NO	357,0309	-0,98
357,0459	5,69	C15H18O6S2	[M - H]-	Yes	NO	Yes	NO	357,0461	-0,58
359,0618	5,76	C15H20O6S2	[M - H]-	Yes	NO	Yes	NO	359,0618	0,12
366,921	6,21	C14H8O4S4	[M - H]-	Yes	NO	NO	NO	366,9222	-3,18
379,0508	5,04	C14H20O8S2	[M - H]-	Yes	Yes	NO	NO	379,0516	-2,07
382,8335	6,85	C5H4O10S5	[M - H]-	Yes	NO	NO	Yes	382,8324	2,80
391,0728	5,52	C12H24O10S2	[M - H]-	Yes	NO	NO	NO	391,0727	0,22
404,9973	12,02	C11H18O10S3	[M - H]-	Yes	NO	Yes	Yes	404,9978	-1,32

Supplementary Table 4: All detected molecular formulas showing a fragmentation pattern in accordance with a sulfonic acid functional group.

Supplementary references

1. Smith, C.A., et al., *XCMS: Processing mass spectrometry data for metabolite profiling using Nonlinear peak alignment, matching, and identification.* Analytical Chemistry, 2006. **78**(3): p. 779-787.
2. Kuhl, C., et al., *CAMERA: an integrated strategy for compound spectra extraction and annotation of liquid chromatography/mass spectrometry data sets.* Anal Chem, 2012. **84**(1): p. 283-9.

3. Franca, R.D.G., et al., *Biodegradation Products of a Sulfonated Azo Dye in Aerobic Granular Sludge Sequencing Batch Reactors Treating Simulated Textile Wastewater.* ACS Sustainable Chemistry & Engineering, 2019. **7**(17): p. 14697-14706.
4. Voogt, P. and M. Saez, *Analytical chemistry of perfluoroalkylated substances.* TrAC Trends in Analytical Chemistry, 2006. **25**(4): p. 326-342.
5. Duhrkop, K., et al., *SIRIUS 4: a rapid tool for turning tandem mass spectra into metabolite structure information.* Nat Methods, 2019. **16**(4): p. 299-302.
