# Supplementary material for: Formation, stabilization and fate of acetaldehyde and higher aldehydes in an autonomously changing prebiotic system emerging from acetylene
Source: Commun Chem. 2023 Feb 22;6:38. doi: 10.1038/s42004-023-00833-5 (PMC9947100; doi:10.1038/s42004-023-00833-5)
Supplement: Supplementary file 2 — Description of Additional Supplementary Files [file 42004_2023_833_MOESM2_ESM.pdf]

Description of Additional Supplementary File

File name: Supplementary Data 1

Description: NMR spectra
