# Supplementary material for: Formation, stabilization and fate of acetaldehyde and higher aldehydes in an autonomously changing prebiotic system emerging from acetylene
Source: Commun Chem. 2023 Feb 22;6:38. doi: 10.1038/s42004-023-00833-5 (PMC9947100; doi:10.1038/s42004-023-00833-5)
Supplement: Supplementary file 3 [file 42004_2023_833_MOESM3_ESM.docx]

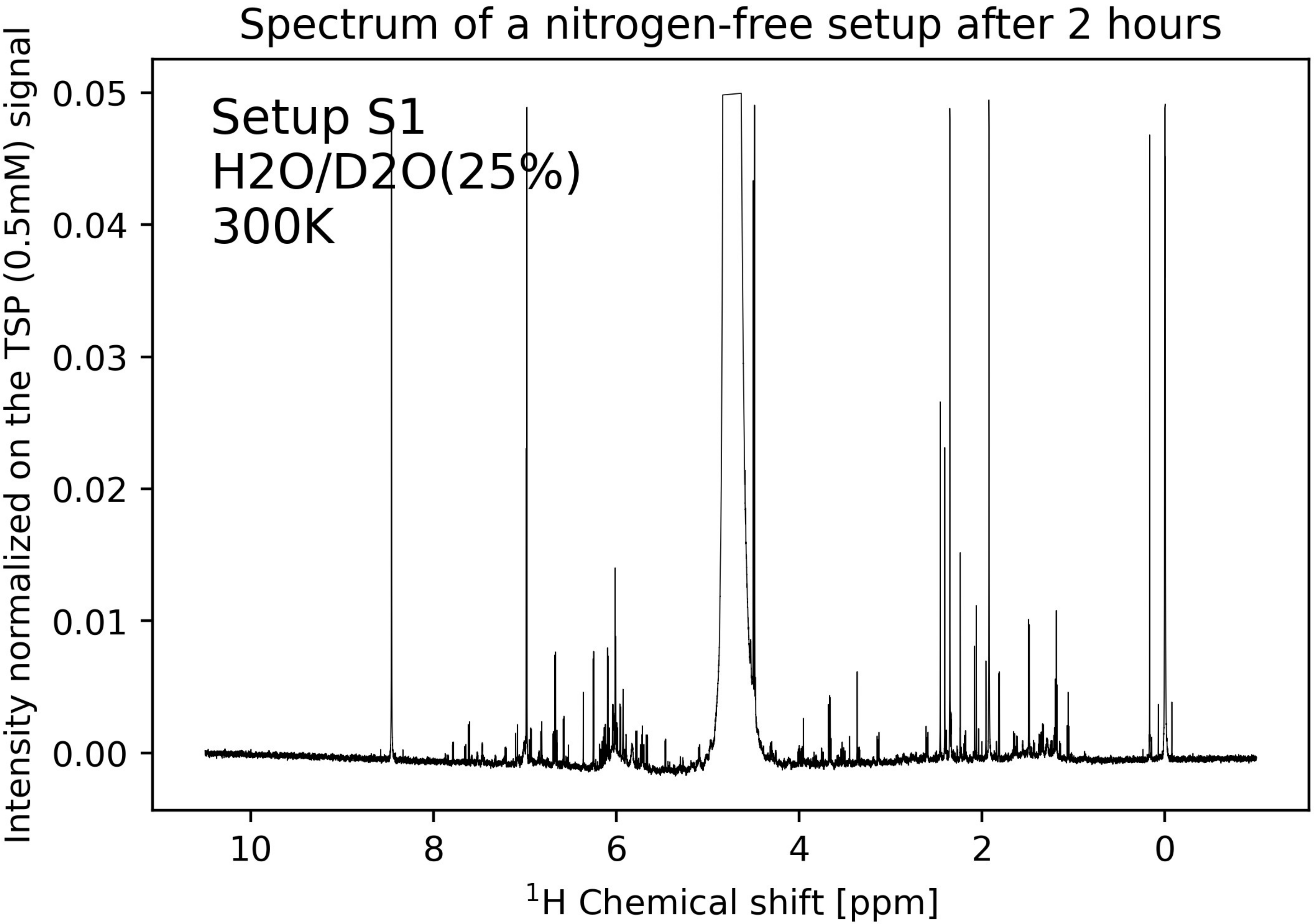
Spectrum of a nitrogen-free setup after 2 hours
Setup S1
H2O/D2O(25%)
300K
Intensity normalized on the TSP (0.5mM) signal
0.05
0.04
0.03
0.02
0.01
0.00
10
8
6
4
2
0
1H Chemical shift [ppm]

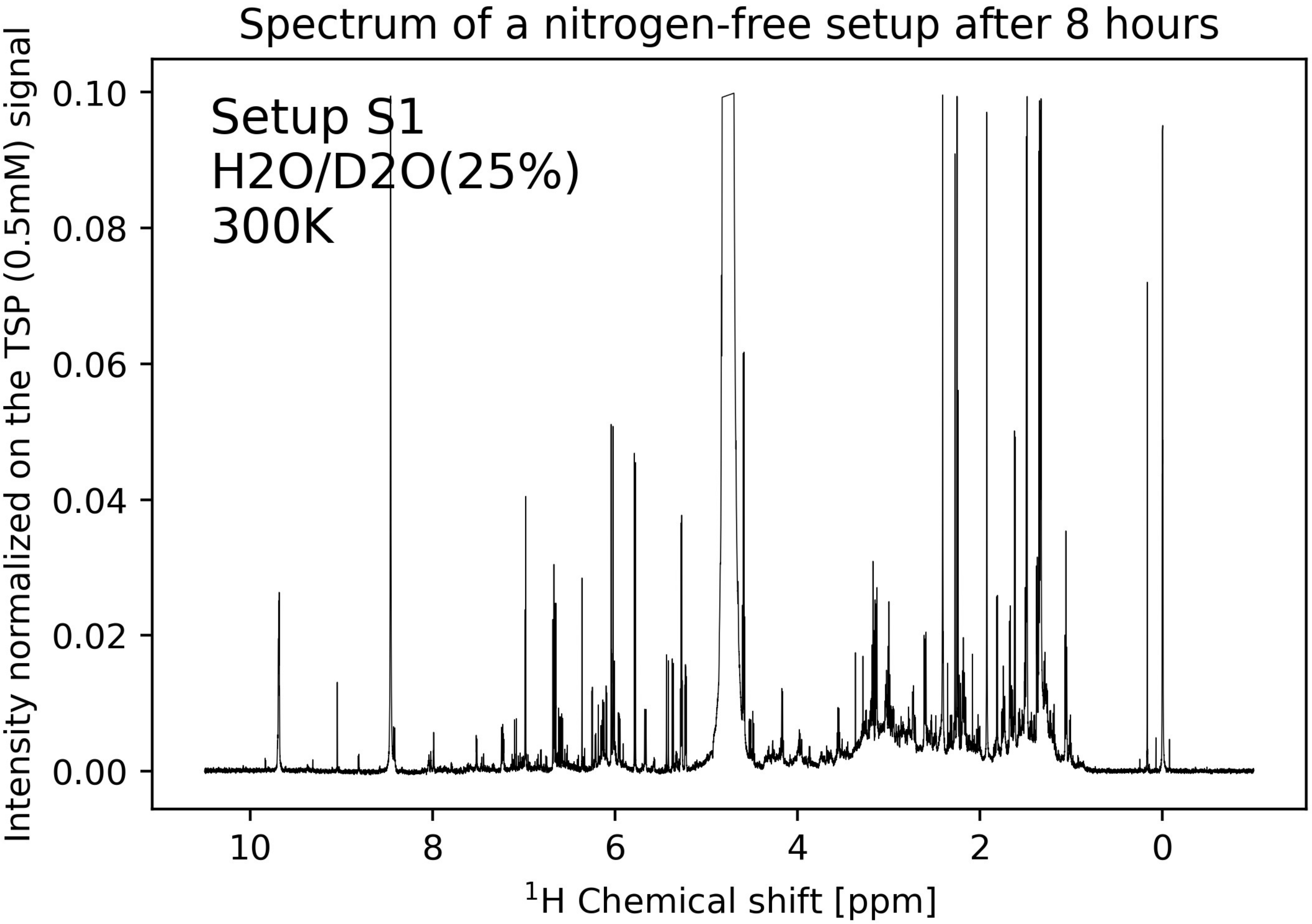
Spectrum of a nitrogen-free setup after 8 hours
Setup S1
H2O/D2O(25%)
300K
Intensity normalized on the TSP (0.5mM) signal
0.00
0.02
0.04
0.06
0.08
0.10
10
8
6
4
2
0
1H Chemical shift [ppm]

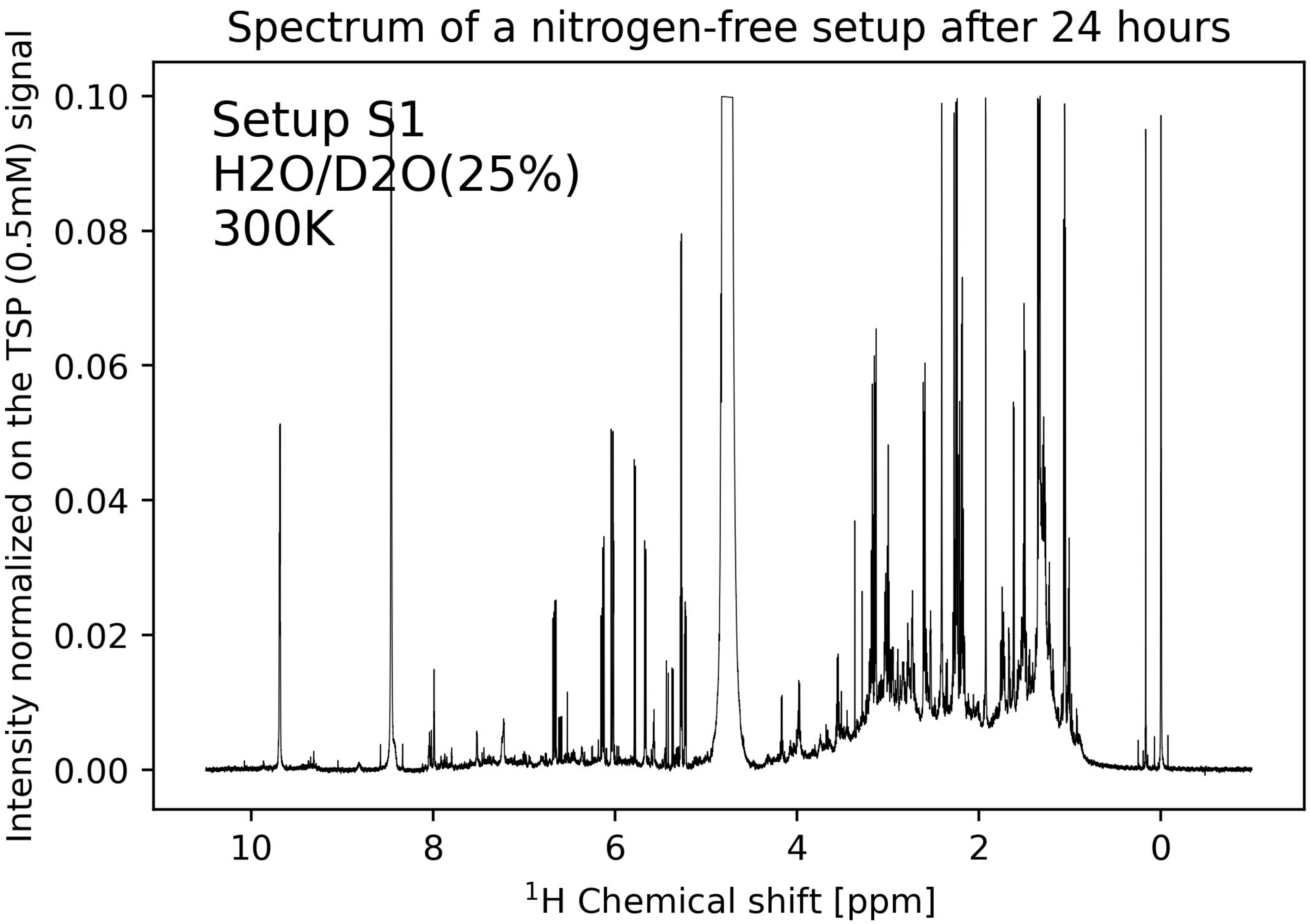

Spectrum of a nitrogen-free setup after 24 hours
Setup S1
H2O/D2O(25%)
300K
Intensity normalized on the TSP (0.5mM) signal
0.00
0.02
0.04
0.06
0.08
0.10
10
8
6
4
2
0
1H Chemical shift [ppm]

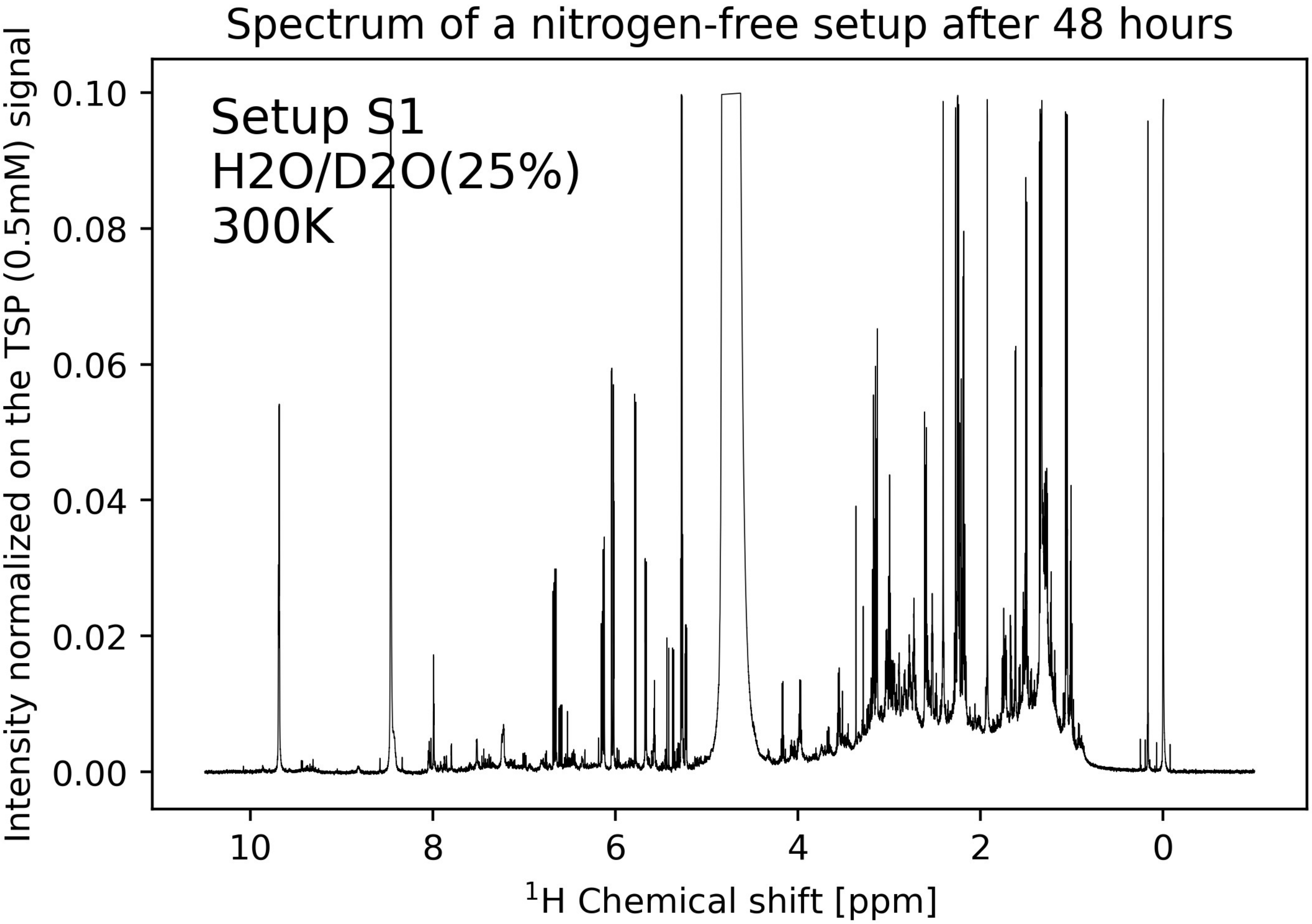
Spectrum of a nitrogen-free setup after 48 hours
Setup S1
H2O/D2O(25%)
300K
Intensity normalized on the TSP (0.5mM) signal
0.00
0.02
0.04
0.06
0.08
0.10
10
8
6
4
2
0
1H Chemical shift [ppm]

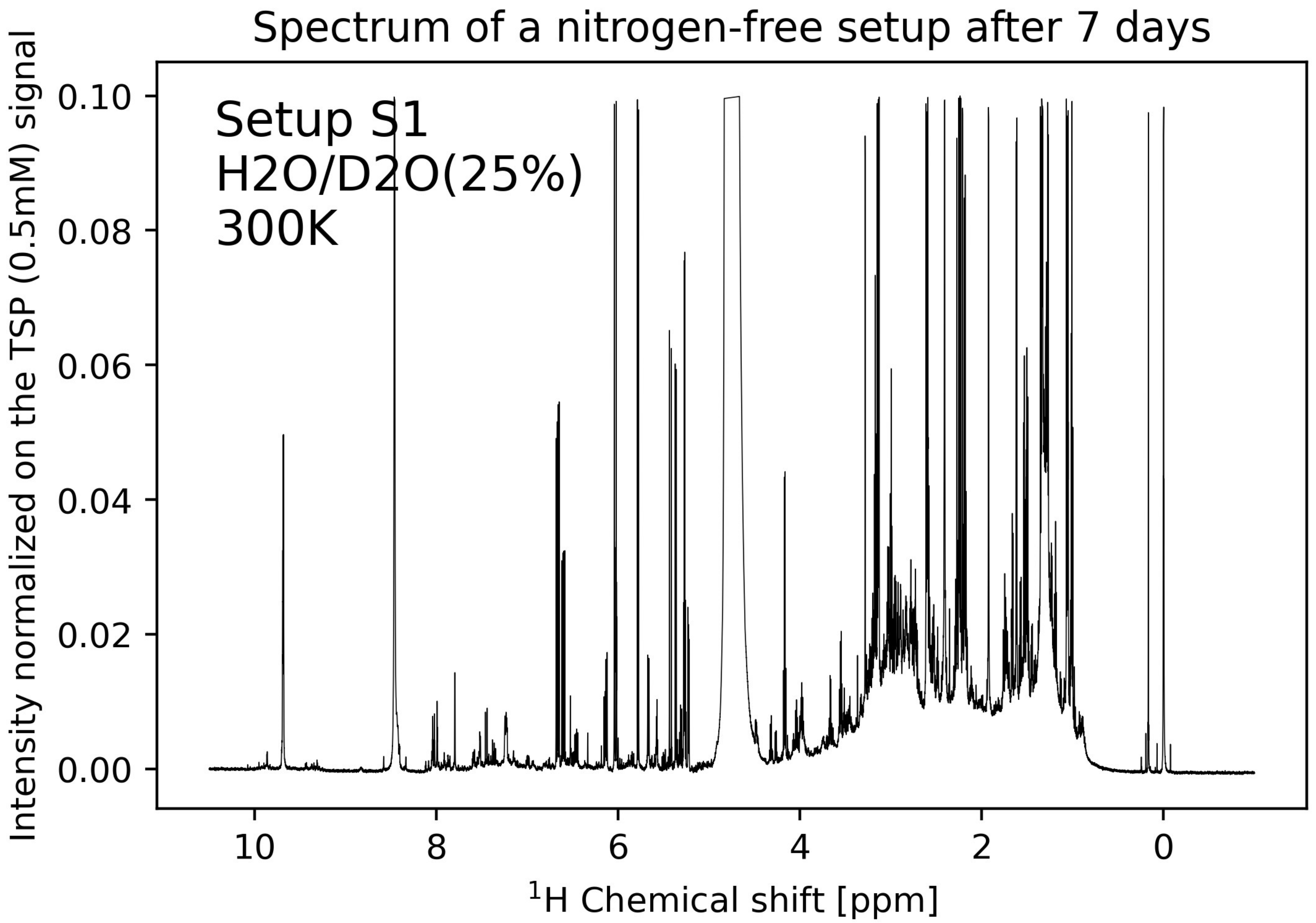

Spectrum of a nitrogen-free setup after 7 days
Setup S1
H2O/D2O(25%)
300K
Intensity normalized on the TSP (0.5mM) signal
0.00
0.02
0.04
0.06
0.08
0.10
10
8
6
4
2
0
1H Chemical shift [ppm]

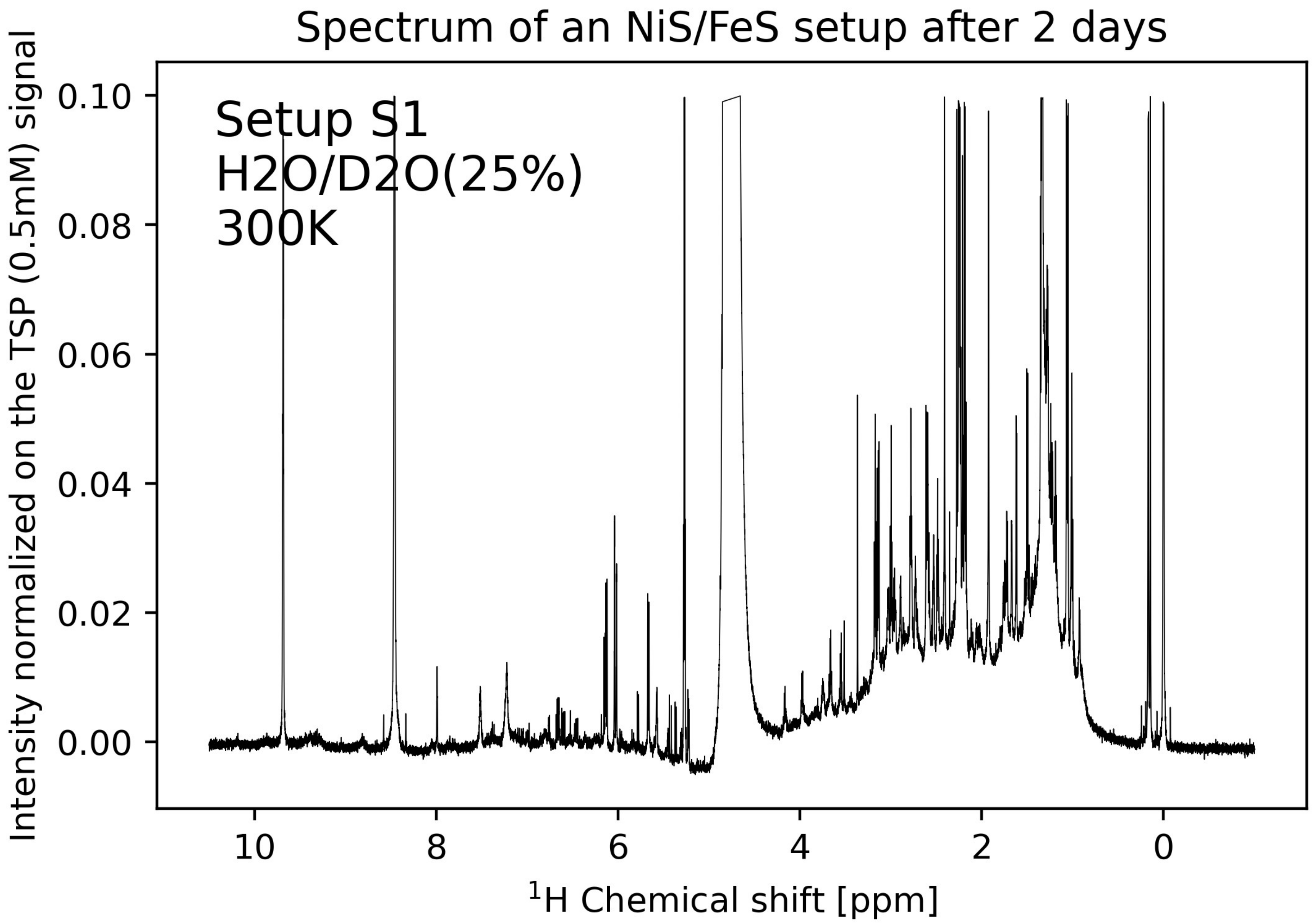

Spectrum of an NiS/FeS setup after 2 days
Setup S1
H2O/D2O(25%)
300K
Intensity normalized on the TSP (0.5mM) signal
0.10
0.08
0.06
0.04
0.02
0.00
10
8
6
4
2
0
1H Chemical shift [ppm]

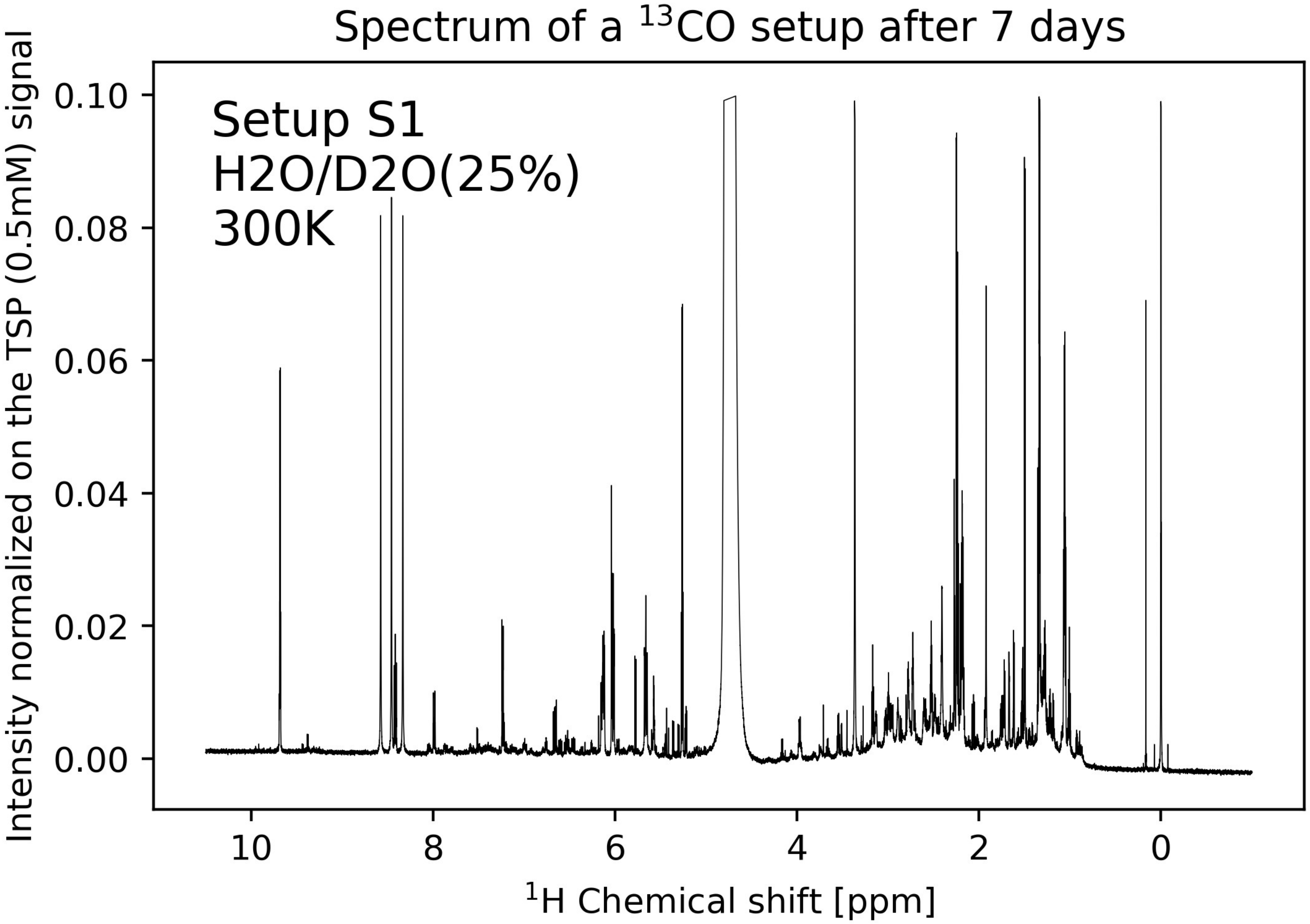
Spectrum of a ^{13}CO setup after 7 days
Setup S1
H2O/D2O(25%)
300K
Intensity normalized on the TSP (0.5mM) signal
0.00
0.02
0.04
0.06
0.08
0.10
10
8
6
4
2
0
^{1}H Chemical shift [ppm]

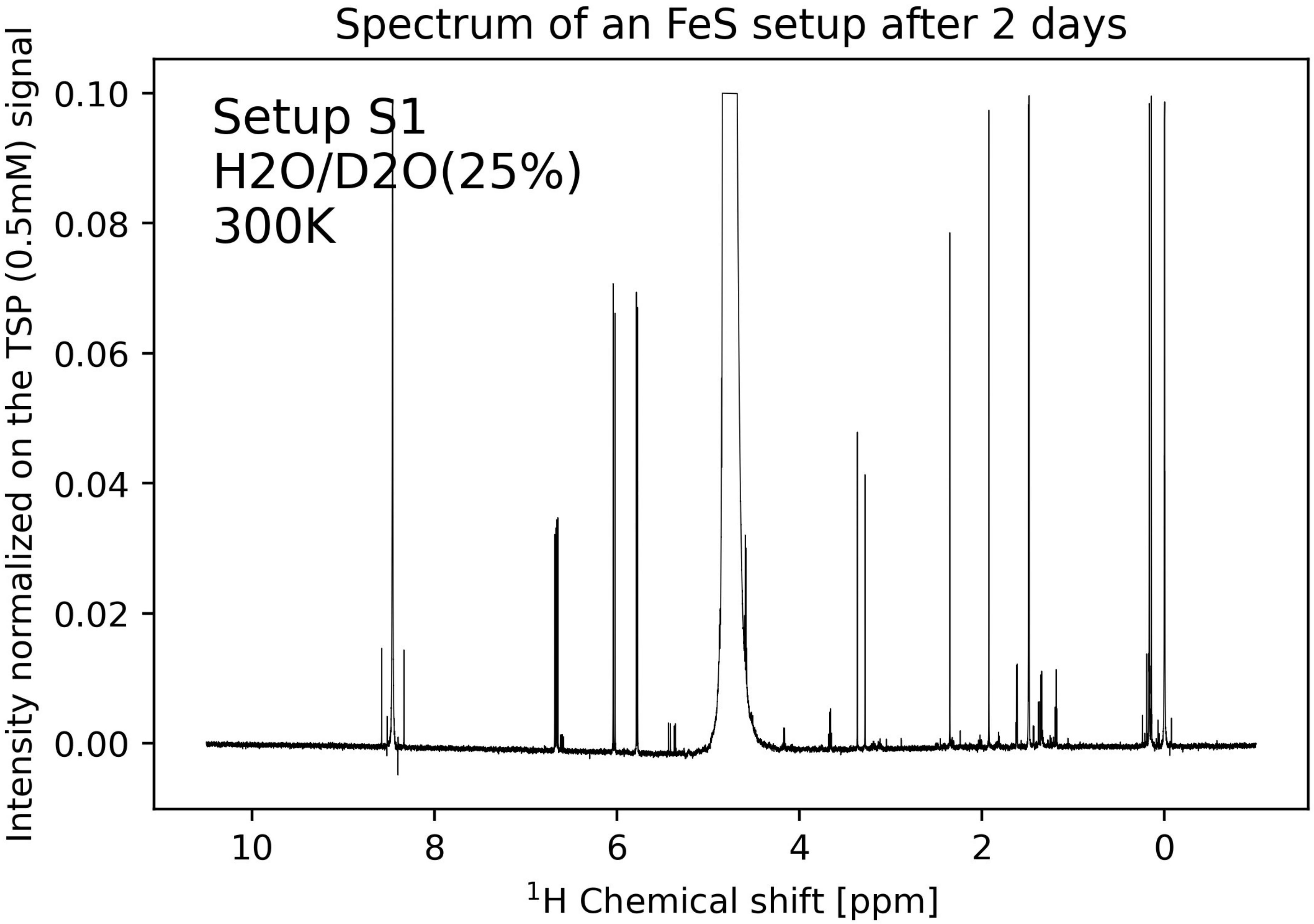

Spectrum of an FeS setup after 2 days
Setup S1
H2O/D2O(25%)
300K
Intensity normalized on the TSP (0.5mM) signal
0.10
0.08
0.06
0.04
0.02
0.00
10
8
6
4
2
0
1H Chemical shift [ppm]

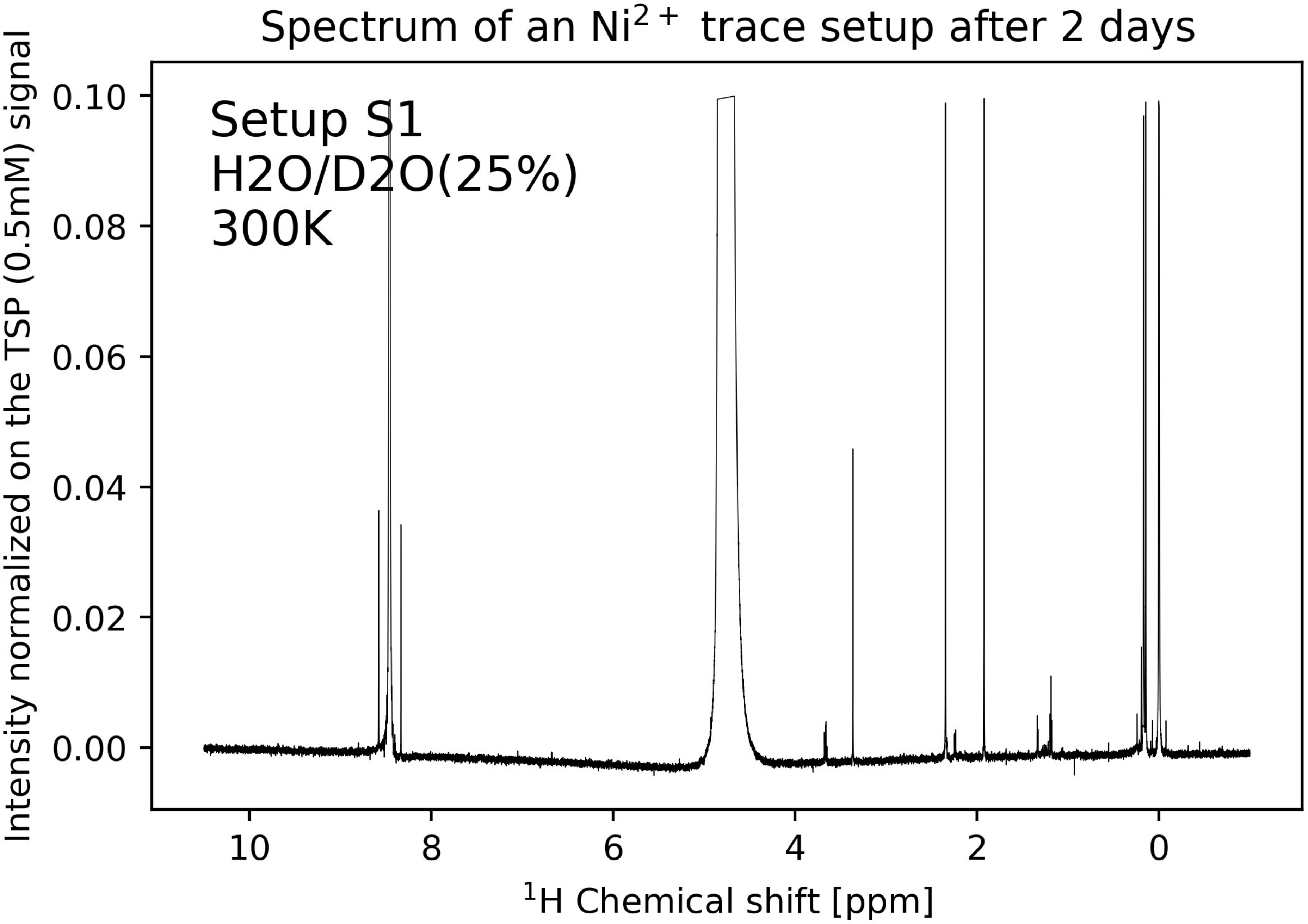

Spectrum of an Ni^{2+} trace setup after 2 days
Setup S1
H2O/D2O(25%)
300K
Intensity normalized on the TSP (0.5mM) signal
0.10
0.08
0.06
0.04
0.02
0.00
10
8
6
4
2
0
^{1}H Chemical shift [ppm]

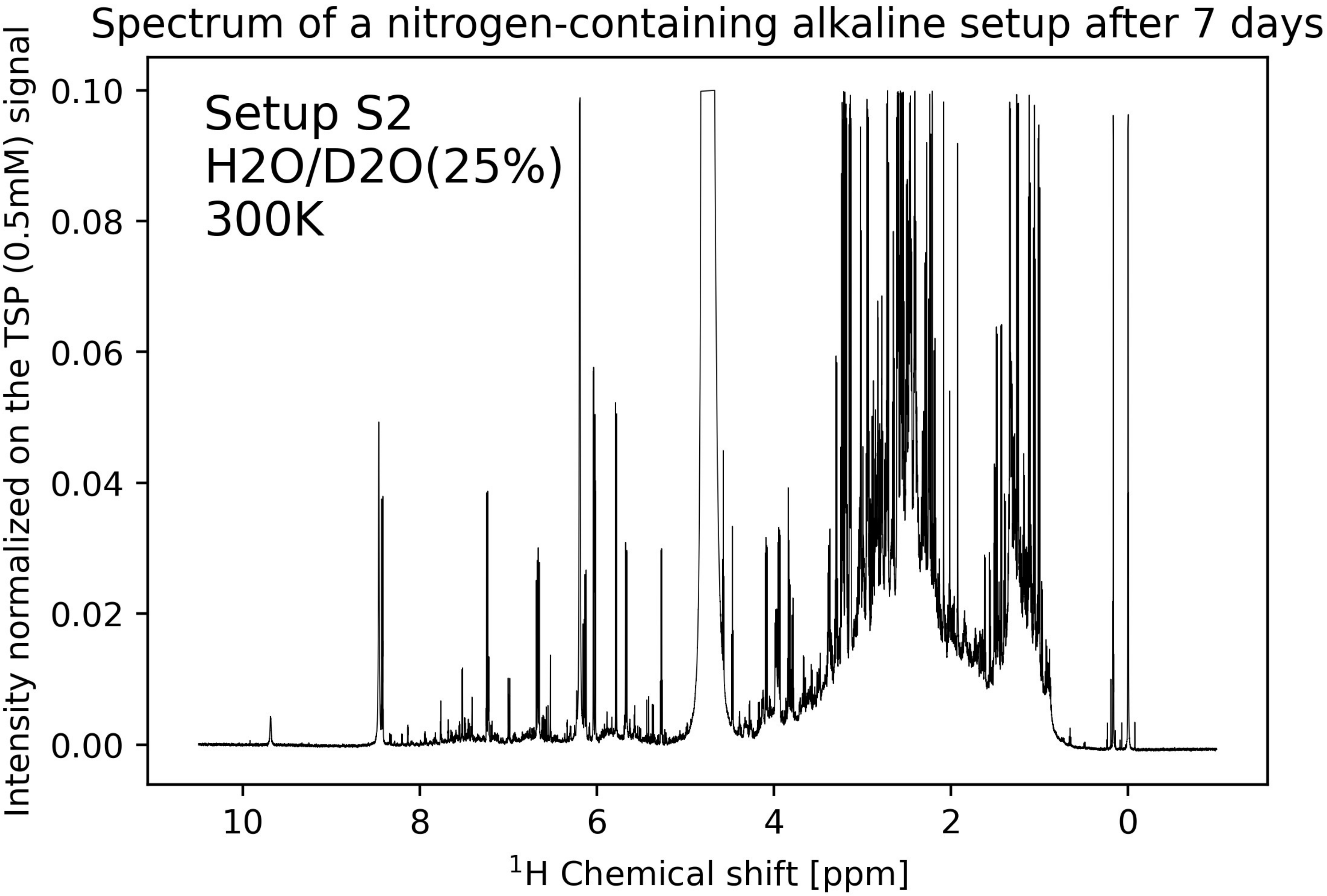
Spectrum of a nitrogen-containing alkaline setup after 7 days
Setup S2
H2O/D2O(25%)
300K
Intensity normalized on the TSP (0.5mM) signal
0.10
0.08
0.06
0.04
0.02
0.00
10
8
6
4
2
0
1H Chemical shift [ppm]

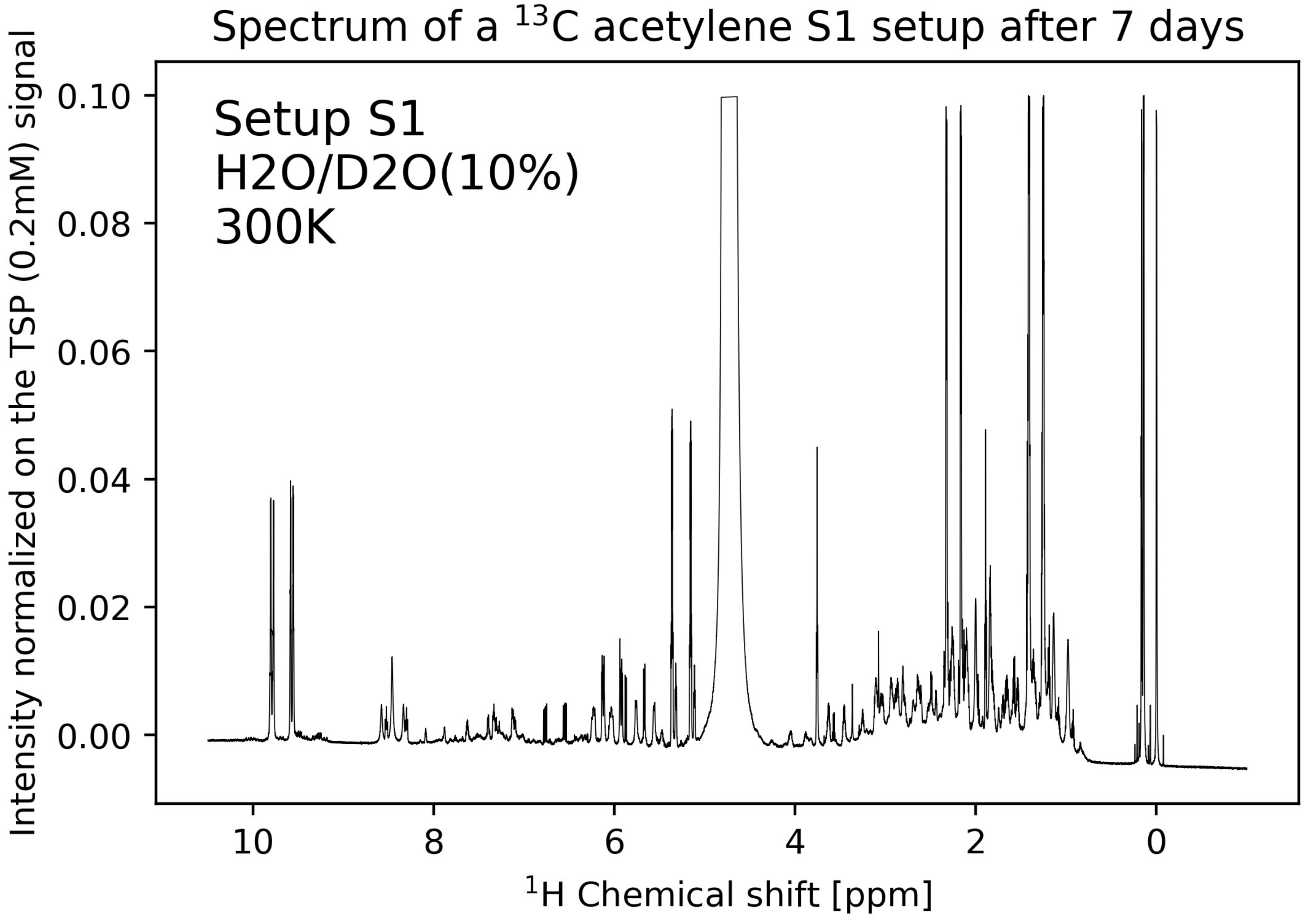

Spectrum of a 13C acetylene S1 setup after 7 days
Setup S1
H2O/D2O(10%)
300K
Intensity normalized on the TSP (0.2mM) signal
0.10
0.08
0.06
0.04
0.02
0.00
10
8
6
4
2
0
1H Chemical shift [ppm]

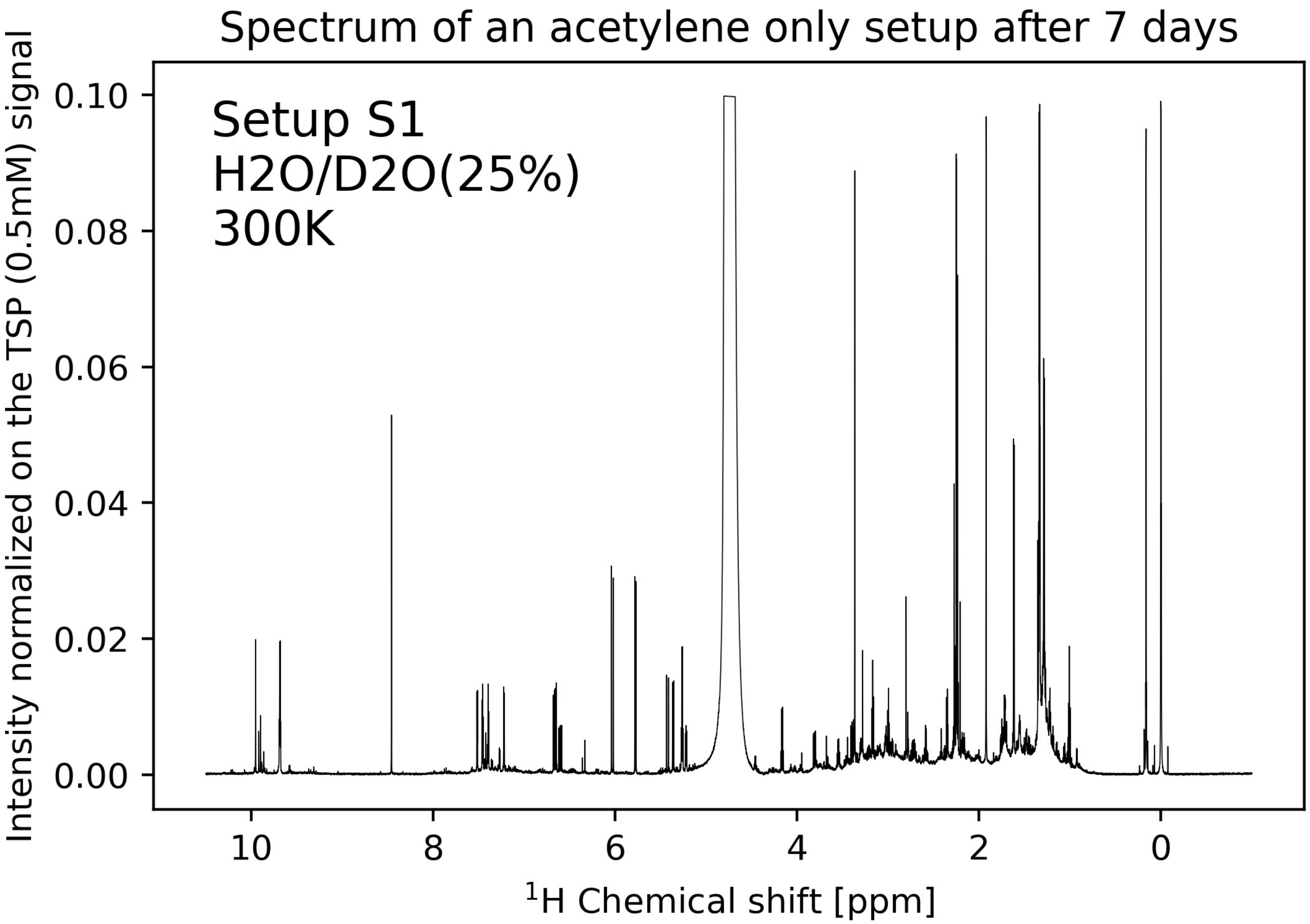

Spectrum of an acetylene only setup after 7 days
Setup S1
H2O/D2O(25%)
300K
Intensity normalized on the TSP (0.5mM) signal
0.00
0.02
0.04
0.06
0.08
0.10
10
8
6
4
2
0
1H Chemical shift [ppm]

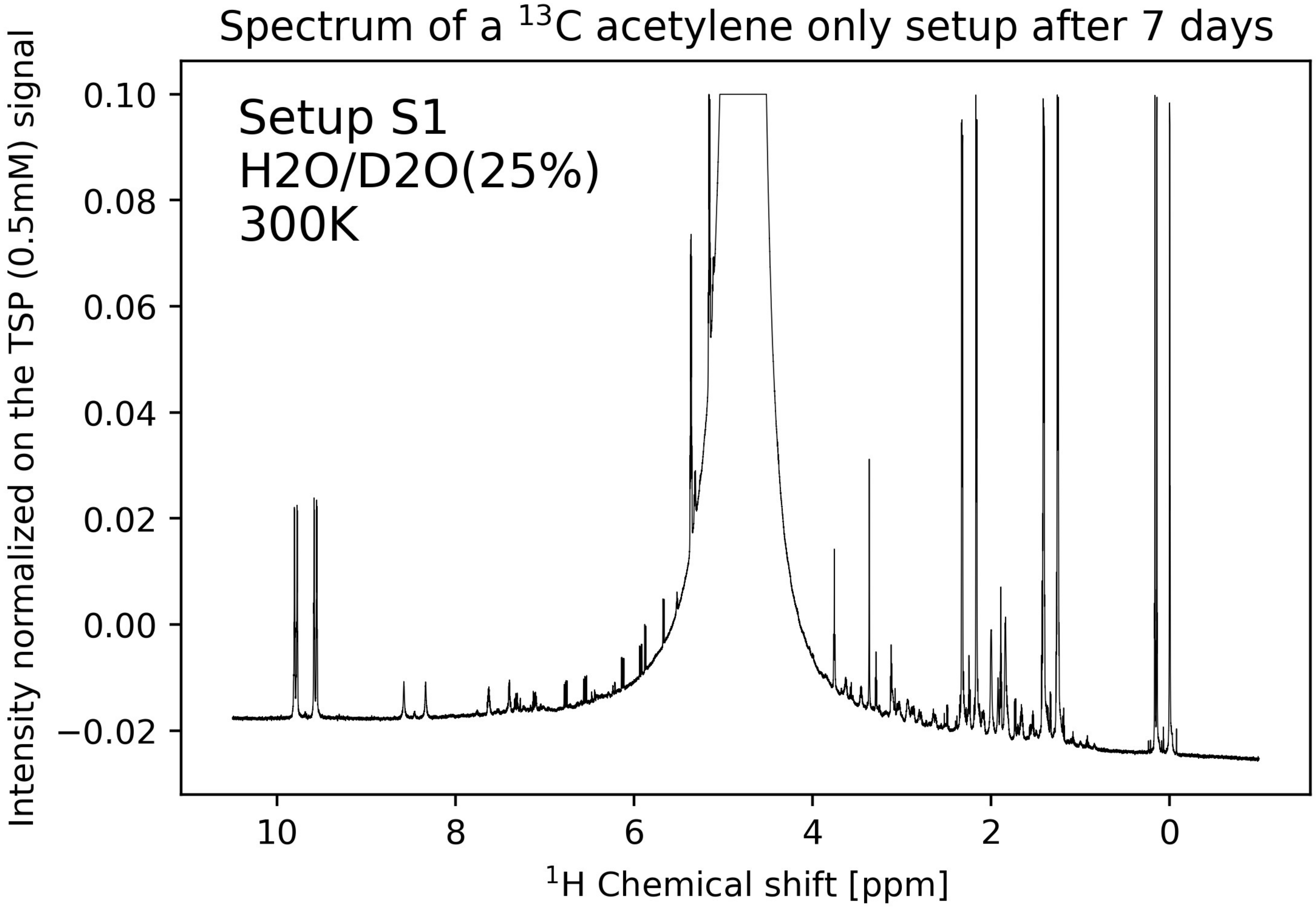

Spectrum of a ^{13}C acetylene only setup after 7 days
Setup S1
H2O/D2O(25%)
300K
Intensity normalized on the TSP (0.5mM) signal
0.10
0.08
0.06
0.04
0.02
0.00
−0.02
10
8
6
4
2
0
^{1}H Chemical shift [ppm]

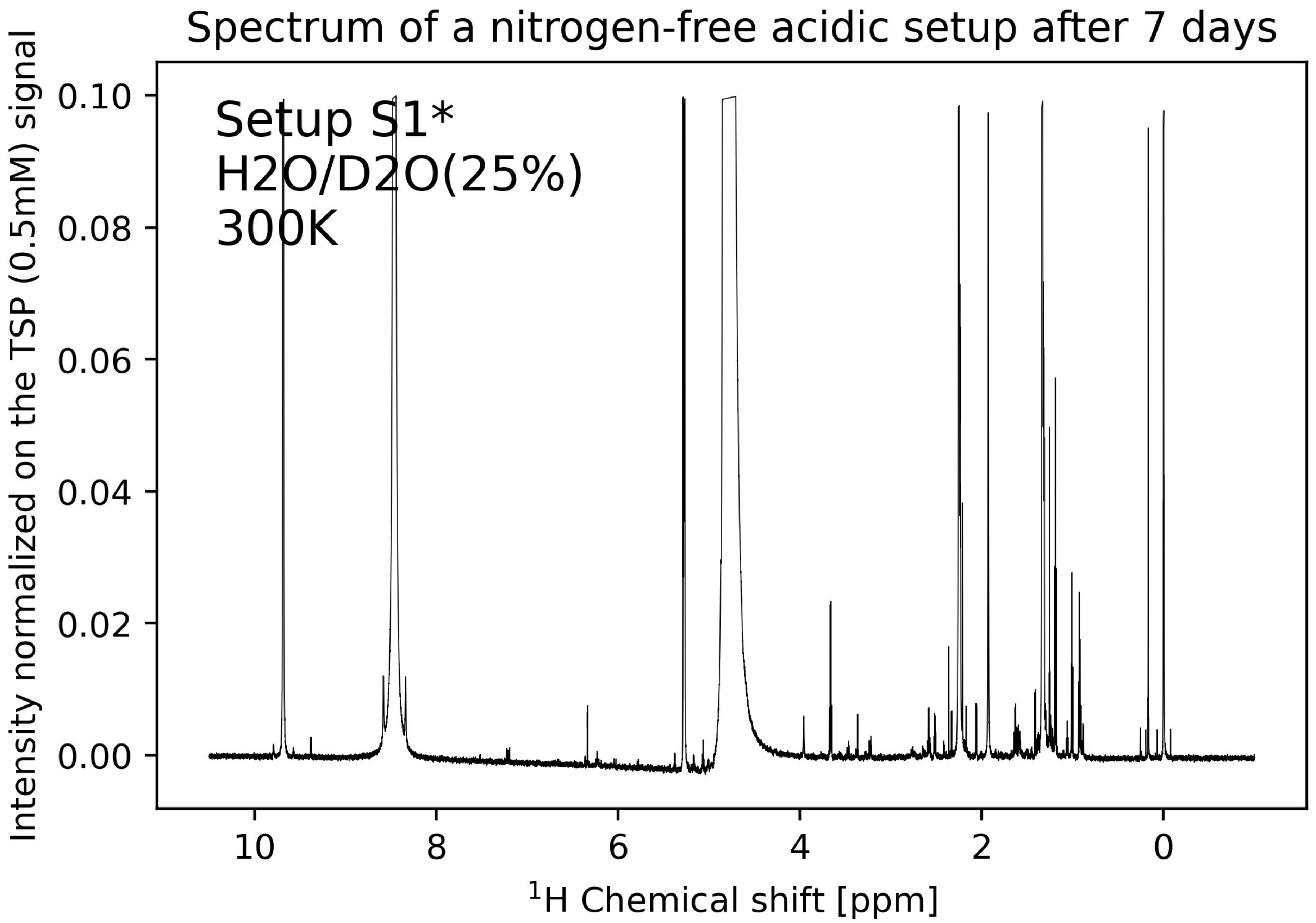

Spectrum of a nitrogen-free acidic setup after 7 days
Setup S1*
H2O/D2O(25%)
300K
Intensity normalized on the TSP (0.5mM) signal
0.10
0.08
0.06
0.04
0.02
0.00
10
8
6
4
2
0
1H Chemical shift [ppm]

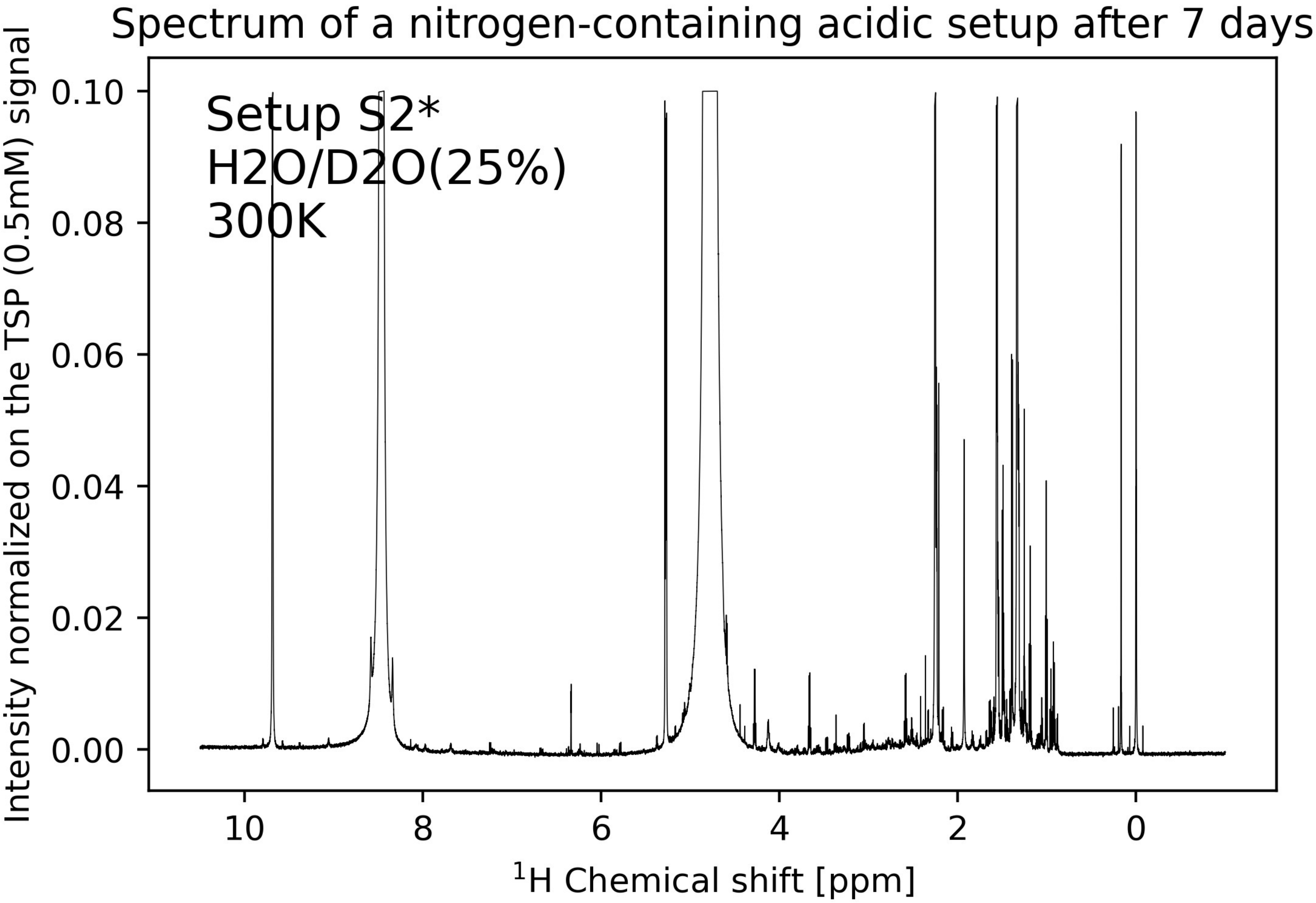

Spectrum of a nitrogen-containing acidic setup after 7 days
Setup S2*
H2O/D2O(25%)
300K
Intensity normalized on the TSP (0.5mM) signal
0.10
0.08
0.06
0.04
0.02
0.00
10
8
6
4
2
0
1H Chemical shift [ppm]

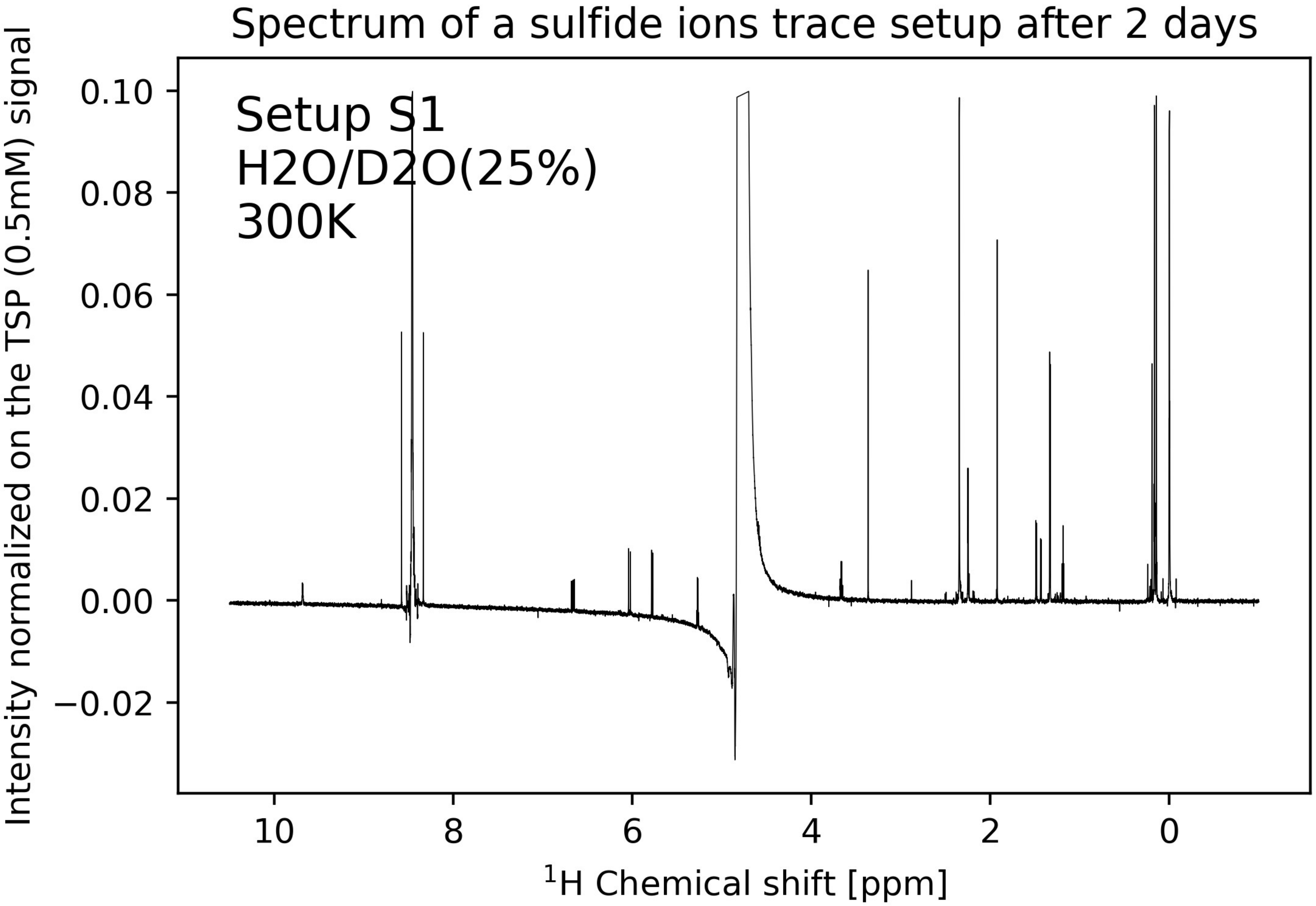
Spectrum of a sulfide ions trace setup after 2 days
Setup S1
H2O/D2O(25%)
300K
Intensity normalized on the TSP (0.5mM) signal
0.10
0.08
0.06
0.04
0.02
0.00
−0.02
10
8
6
4
2
0
1H Chemical shift [ppm]
